# Combined Drug Action of 2-Phenylimidazo[2,1-*b*]Benzothiazole Derivatives on Cancer Cells According to Their Oncogenic Molecular Signatures

**DOI:** 10.1371/journal.pone.0046738

**Published:** 2012-10-05

**Authors:** Alessandro Furlan, Benjamin Roux, Fabienne Lamballe, Filippo Conti, Nathalie Issaly, Fabrice Daian, Jean-François Guillemot, Sylvie Richelme, Magali Contensin, Joan Bosch, Daniele Passarella, Oreste Piccolo, Rosanna Dono, Flavio Maina

**Affiliations:** 1 Aix-Marseille Univ, IBDML, CNRS UMR 7288, Marseille, France; 2 Laboratory of Organic Chemistry, Faculty of Pharmacy and Institute of Biomedicine (IBUB), University of Barcelona, Barcelona, Spain; 3 Dipartimento di Chimica Organica e Industriale, Università degli Studi di Milano, Milano, Italy; 4 Studio di consulenza scientifica, Sirtori (LC), Italy; Ludwig-Maximilians University, Germany

## Abstract

The development of targeted molecular therapies has provided remarkable advances into the treatment of human cancers. However, in most tumors the selective pressure triggered by anticancer agents encourages cancer cells to acquire resistance mechanisms. The generation of new rationally designed targeting agents acting on the oncogenic path(s) at multiple levels is a promising approach for molecular therapies. 2-phenylimidazo[2,1-*b*]benzothiazole derivatives have been highlighted for their properties of targeting oncogenic Met receptor tyrosine kinase (RTK) signaling. In this study, we evaluated the mechanism of action of one of the most active imidazo[2,1-*b*]benzothiazol-2-ylphenyl moiety-based agents, Triflorcas, on a panel of cancer cells with distinct features. We show that Triflorcas impairs in vitro and in vivo tumorigenesis of cancer cells carrying Met mutations. Moreover, Triflorcas hampers survival and anchorage-independent growth of cancer cells characterized by “RTK swapping” by interfering with PDGFRβ phosphorylation. A restrained effect of Triflorcas on metabolic genes correlates with the absence of major side effects in vivo. Mechanistically, in addition to targeting Met, Triflorcas alters phosphorylation levels of the PI3K-Akt pathway, mediating oncogenic dependency to Met, in addition to Retinoblastoma and nucleophosmin/B23, resulting in altered cell cycle progression and mitotic failure. Our findings show how the unusual binding plasticity of the Met active site towards structurally different inhibitors can be exploited to generate drugs able to target Met oncogenic dependency at distinct levels. Moreover, the disease-oriented NCI Anticancer Drug Screen revealed that Triflorcas elicits a unique profile of growth inhibitory-responses on cancer cell lines, indicating a novel mechanism of drug action. The anti-tumor activity elicited by 2-phenylimidazo[2,1-*b*]benzothiazole derivatives through combined inhibition of distinct effectors in cancer cells reveal them to be promising anticancer agents for further investigation.

## Introduction

Receptor tyrosine kinase (RTK) signaling has been implicated in tumor evolution for its capacity to influence cell fate through changes in key regulatory circuits [1]–[3]. As evidenced by cancer genomic studies, RTK signaling is one core pathway frequently altered in human cancer [4], [5]. We have recently shown the relevance of signaling nodes interconnecting RTK and p53 core pathways and the impact of targeting such nodes during tumor evolution [6], [7]. The relevance of altered RTKs in oncogenesis has drawn tremendous interest to identify agents capable of restraining their activity and function. To date, molecular therapies for “RTK-addicted” cancer cells are mainly based on the application of compounds that selectively target the oncogenic RTK [1]. However, the success of these strategies has been limited since inhibition of the “primary RTK-addiction” triggers a selective pressure on cancer cells to acquire resistance through “RTK swapping” [8], [9]. These limitations impose the identification, or the combined use, of agents that not only target RTK signaling dependency, but also hamper adaptation caused by redundancy in the RTK signaling network [10].

One approach to circumvent “RTK swapping” could be the identification of drugs interfering with oncogene dependency by acting at multiple levels within the addiction path. An example of this concept is provided by Sorafenib, a small chemical agent able to inhibit several RTKs, including VEGFR, PDGFRβ, Kit, FGFR1, Ret, and the intracellular Raf kinase [11]. The broad-spectrum activity of Sorafenib in several cancer models is likely due to the wide range of its targets. Nevertheless, Sorafenib activity in some types of tumor models is attributed to the concomitant inhibition of RTK-driven angiogenesis and the RTK downstream Raf/MAPK pathway [11]. The generation of agents, which target oncogenic path(s) at multiple levels, is not a simple issue as distinct targets require a precise drug structure and chemical modifications of drugs can either affect selectivity (e.g. by targeting multiple RTKs), effectiveness, or toxcicity. In contrast to other RTKs, the hepatocyte growth factor (HGF) receptor Met is characterized by unusual structural plasticity as its active site can adopt distinct inhibitor binding modes [12]. Indeed, a wide range of small-molecules have been discovered as Met inhibitors [13], [14]. Nevertheless, efforts continue to uncover novel anti-Met agents for targeted therapies and associated resistance mechanisms [8], [15]–[17].

To identify chemical agents capable of inhibiting oncogenic Met signaling in cancer cells, we previously applied a Met-focused cell-based screen. We had reasoned that such a strategy would offer the possibility of identifying compounds that may: a) elicit inhibitory effects directly on Met; b) target other essential components in the Met signaling cascade; c) be well tolerated due to limited toxic effects at biologically active concentrations [18]–[20]. We reported that new amino acid amides containing the imidazo[2,1-*b*]benzothiazol-2-ylphenyl moiety target Met directly and inhibit oncogenic Met function, without eliciting major side effects in vitro [19]. In this study, we explored the anticancer activity of one of the most active agents we have identified, Triflorcas (TFC), on a panel of cancer cells with distinct characteristics and investigated its mechanism of drug action by a range of complementary approaches. We show that Triflorcas targets cancer cells either carrying Met mutations or characterized by RTK swapping. We demonstrate that Triflorcas is well tolerated in vivo and does not significantly alter the expression of several cell toxicity and stress genes. Biochemical and phospho-screening array studies revealed that Triflorcas predominantly alters the phosphorylation levels of the PI3K/Akt pathway, which ensures oncogenic dependency to Met, as well as Retinoblastoma (Rb), and nucleophosmin/B23. These alterations functionally correlate with changes in cell cycle progression underlying mitotic failure. Although Triflorcas anticancer activity correlates with its inhibitory effects on Met, its drug action mechanisms may not be merely restricted to Met target itself. The unique ability of Triflorcas to modulate multiple pathways deregulated in tumor cells with aberrant Met signaling further strengthens the prospect of exploiting the flexible-binding mode capacity of Met active site to identify new agents with inhibitory properties towards signaling targets required to execute the oncogenic program. Finally, the assessment of the inhibitory-response profile on cancer cells through the National Cancer Institute anticancer drug screen suggests that Triflorcas is characterized by a novel mechanism of drug action. Bioinformatics studies indicate possible molecular signatures that correlate with cancer sensitivity to imidazo[2,1-*b*]benzothiazol-2-ylphenyl moiety-based agents.

## Results

### Triflorcas Inhibits Survival and Anchorage-independent Growth of Human Cancer Cells Carrying Mutated Met

**Figure 1:**
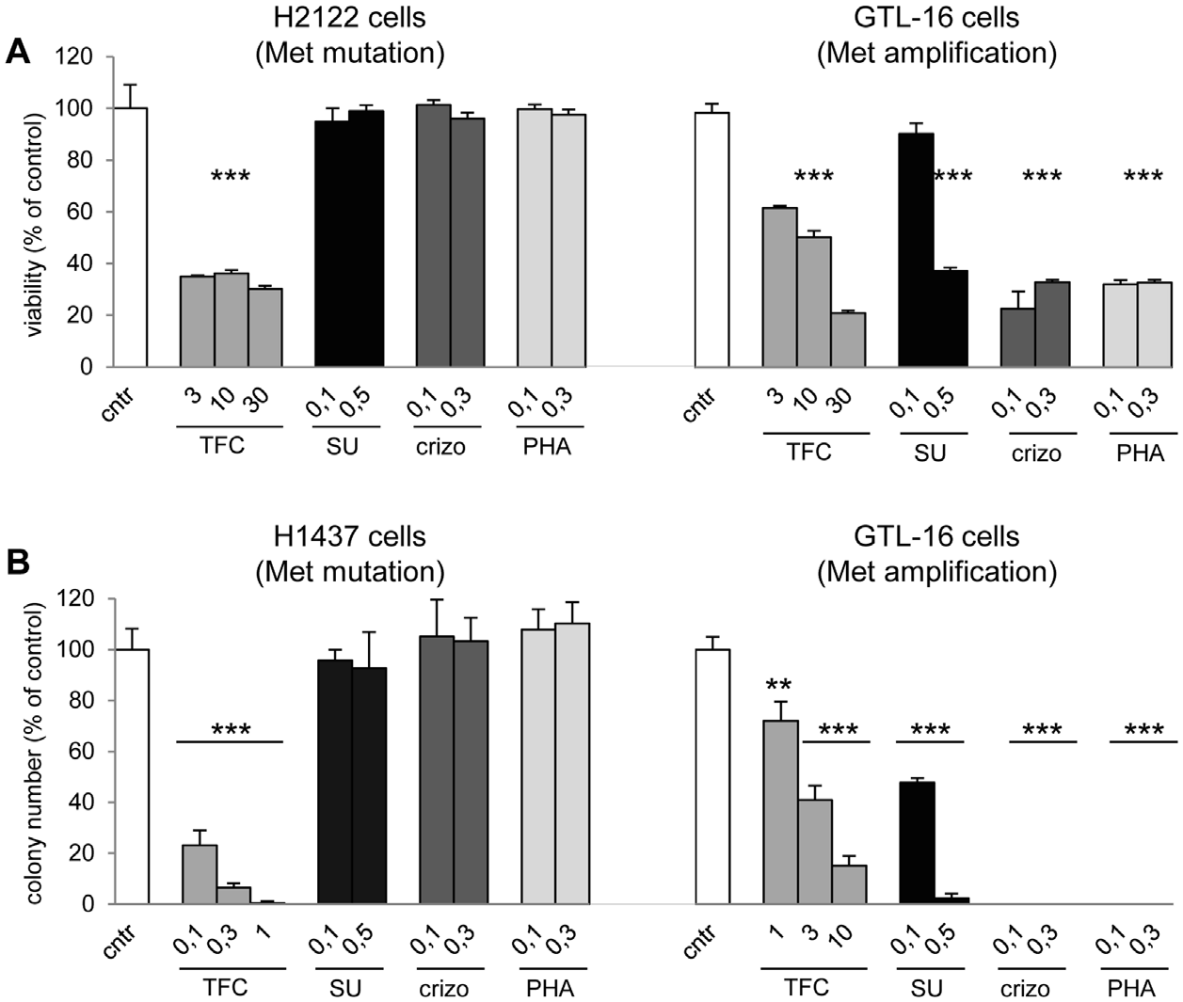
Triflorcas blocks Met-triggered cell survival and in vitro tumorigenesis of human NSCLC cells carrying Met mutations (H2212 and H1437) and of human gastric carcinoma cells carrying Met amplification (GTL-16). (A) Survival of H2122 and GTL-16 cells was reduced by Triflorcas at indicated concentration (µM; n = 3). In contrast, SU11274 (SU), crizotinib (crizo), and PHA665752 (PHA) impaired survival of GTL-16, but not of H2212 cells. Cells were serum-starved for 24 hours and then incubated with compounds for 48 hours. (B) Triflorcas blocked anchorage-independent growth of H1437 and GTL-16 cells in a dose dependent manner (n = 3). SU11274, crizotinib, and PHA665752 impaired in vitro tumorigenesis of GTL-16, but not of H1437 cells. Values are expressed as means ± s.e.m. **P<0.01; ***P<0.001; Student-*t* test.

We first examined the effect of Triflorcas on two human non-small-cell lung cancer (NSCLC) cells carrying Met mutations: H2122 and H1437 cells, harboring point mutations at the amino acid residue N375S and R988C, respectively [21]. Triflorcas impaired survival and anchorage independent growth of H2122 and H1437, respectively (Figure 1A and B). None of the Met inhibitors used as reference compounds, such as SU11274, crizotinib, and PHA665752 interfered with survival and in vitro tumorigenesis of these cells (Figure 1A and B). In contrast, all tested inhibitors impaired survival and anchorage independent growth of human gastric carcinoma GTL-16 characterized by Met amplification (Figure 1A and B), as previously reported [19], [21]. These data suggest that Triflorcas exerts a marked inhibitory effect on cancer cells with Met mutations, which are not sensitive to other Met inhibitors, in addition to cells carrying Met amplification.

### Triflorcas Interferes with Met Phosphorylation, with Met Localization, and with PI3K-Akt Pathway Activation

**Figure 2:**
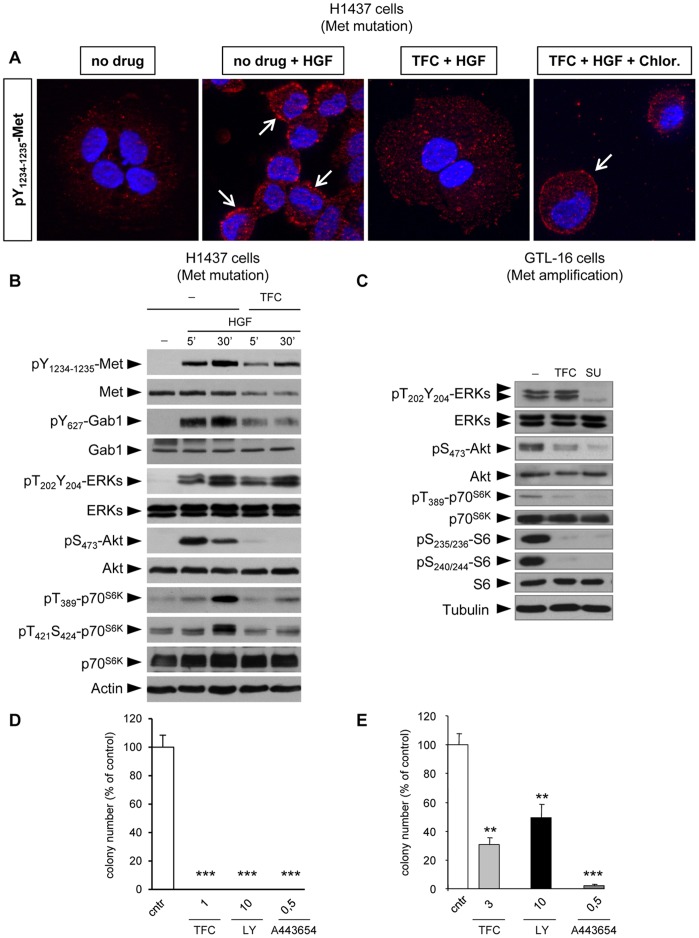
Triflorcas interferes with Met phosphorylation, its cellular localization, and activation of the PI3K/Akt pathway. (A) Met phosphorylation was analyzed by immuno-cytochemistry on H1437 cells untreated, treated with HGF, with Triflorcas (TFC; 10 µM for 24 hours) or with Triflorcas (10 µM for 24 hours) plus chlorpromazine (Chlor; 10 µg/ml for 2 hours) followed by HGF stimulation (20 ng/ml for 30 minutes). Triflorcas reduced the levels of Met phosphorylation induced by HGF. Note also that HGF-induced phospho-Met is localized at the plasma membrane of control cells, whereas it appears internalized in cells exposed to Triflorcas. Endocytosis inhibition with the chlorpromazine drug restored phospho-Met localization at the cellular membrane. Arrows indicate cluster of phosphorylated Met at the plasma membrane (40X magnification). (B) HGF-induced (20 ng/ml) phosphorylation levels of Met, Gab1, Akt, and p70^S6K^ were reduced in H1437 cells exposed to Triflorcas. In contrast, no changes were observed on ERKs phosphorylation levels. Similar expression levels of total Gab1, Akt, and p70^S6K^ were also found, indicating that Triflorcas interfered with their phosphorylation rather than with their expression levels. Western blot analyses were performed on total protein lysates. (C) Phosphorylation levels of Akt, p70^S6K^, and S6 ribosomal protein, but not ERKs, were reduced in GTL-16 cells exposed to Triflorcas. Actin or Tubulin protein levels were used as loading controls in all experiments (lower panels in B and C). (D and E) Anchorage-independent growth of H1437 (D) and of GTL-16 (E) cells was impaired in the presence of Triflorcas (TFC), LY294002 (PI3K inhibitor), or A443654 (Akt inhibitor). Values are expressed as means ± s.e.m. **P<0.01; ***P<0.001; Student-*t* test.

We have previously shown that Triflorcas interferes with Met phosphorylation in living cells and with Met activation in vitro [19]. We therefore investigated the effects of Triflorcas on Met in H1437 cells by following Met phosphorylation on two tyrosine residues located in its kinase domain, Tyr_1234_ and Tyr_1235_. Immunocytochemical analysis revealed Met phosphorylation predominantly on the plasma membrane when H1437 cells were exposed to HGF stimulation (Figure 2A). Notably, we found that Triflorcas leads to changes in phosphorylated Met: a) down-regulation of its phosphorylation levels and b) a predominant localization in intracellular compartments (Figure 2A). Treatment with chlorpromazine, a cationic amphipathic drug that inhibits clathrin-mediated endocytosis, restored phospho-Met localization at the cellular membrane, thus indicating that Triflorcas enhances Met internalization (Figure 2A) Reduced Met phosphorylation was also observed in protein lysates from H1437 cells accompanied by reduced Met protein levels (Figure 2B). Densitometric analysis indicated that Met down-regulation through endocytosis causes the decrease in Met phosphorylation (data not shown). Consistently, we found reduced phosphorylation of Gab1, which is an immediate signaling target of Met (Figure 2B).

The biological effect of Triflorcas on cells carrying Met mutations and Met amplification (Figure 1) [19] led us to evaluate the phosphorylation status of RTK downstream effectors. Among pathways required in cancer cells with oncogenic Met, it has been shown that only a subset of Met-activated pathways sustains the dependency of cancer cells on Met. In particular, the Ras/ERKs and the PI3K/Akt are two pathways that predominantly ensure dependence on oncogenic Met [22]. We therefore explored whether Triflorcas restricts the activation of these two pathways by following the phosphorylation level of ERKs and Akt in H1437 cells. No significant changes were observed on HGF-induced ERK phosphorylation when H1437 cells were exposed to Triflorcas (Figure 2B). In contrast, Akt phosphorylation was significantly reduced after Triflorcas treatment (Figure 2B). Reduced phospho-Akt was paralleled with a decrease in the phosphorylation levels of its downstream signal p70^S6K^ (Figure 2B). Consistently, reduced phosphorylation levels of Akt and its downstream signals p70^S6K^ and S6 ribosomal protein, but not ERKs, were observed also in GTL-16 cells (Figure 2C). We confirmed the functional relevance of intact PI3K/Akt signaling for anchorage-independent growth of H1437 and GTL-16 cells by pharmacologically blocking its activation with LY294002 (PI3K inhibitor) or A-443654 (Akt inhibitor) (Figure 2D, E, S1A, and 1B). The decrease of Akt phosphorylation after Triflorcas treatment was also observed in the ErbB1-addicted human breast cancer BT474 cells, where ErbB1 phosphorylation levels were unchanged (Figure S1C) [19]. Together, these results indicate that the reduction of PI3K/Akt pathway activation by Triflorcas is not merely a consequence of the inhibition of upstream RTK activity. We also found that Akt activity is not required for survival of BT474 cells (Figure S1D). These results provide insights into BT474 cell resistance to Triflorcas treatment by showing that their addiction to oncogenic ErbB1 is ensured by pathway(s) other than PI3K/Akt. Together, these findings reveal that the anti-tumor activity elicited by Triflorcas occurs through combined outcomes on distinct effectors involved in RTK-driven oncogenic dependency.

### Triflorcas Impairs in vivo Tumor Growth of Human Cancer Cells Carrying Met Mutation, Without Causing Major Side Effects

**Figure 3:**
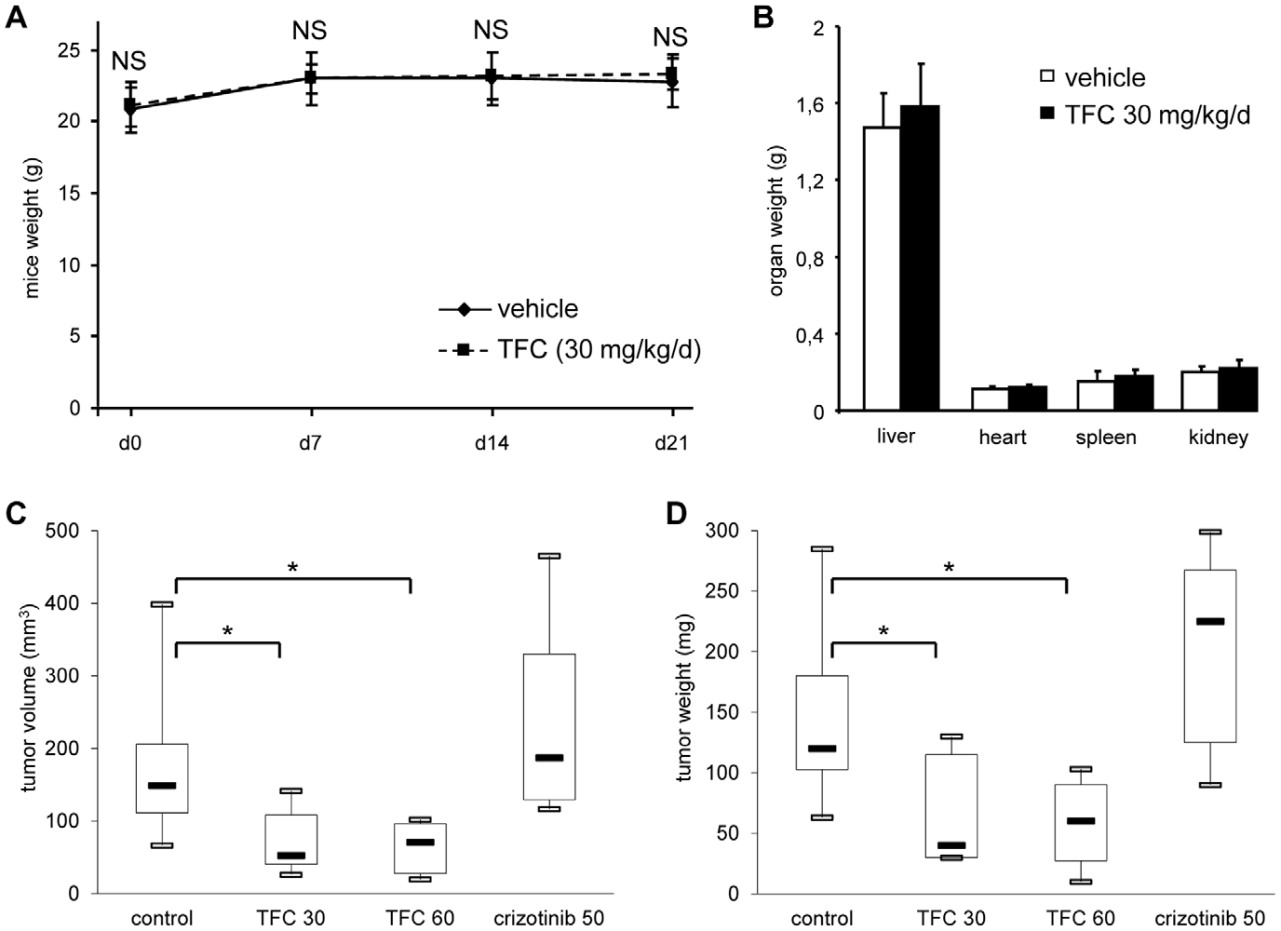
Triflorcas impairs in vivo tumor growth of cancer cells carrying oncogenic Met, without causing major side effects. (A) Evolution of the body weight in mice, treated intra-peritoneally with either vehicle or Triflorcas (TFC; 30 mg.kg^−1^ every day) showed no significant differences. Body weight is expressed as weight evolution over the 21 day-treatment period. (B) The weight of heart, spleen, kidney, and liver was evaluated in mice daily injected with Triflorcas (30 mg.kg^−1^) or vehicle for 21 days. No significant differences were observed. Values are expressed as means ± s.e.m. *P*>0.05. (C and D) Triflorcas treatment (i.p. 30 and 60 mg.kg^−1^ every other day) reduced tumor volume (C) and weight (D) in nude mice injected sub-cutaneously with H1437 cells. Crizotinib (50 mg.kg^−1^ daily) did not impair tumor growth. Values are reported as boxplots and expressed as means ± s.e.m. *P<0.05; Student-*t* test.

We have recently reported that Triflorcas is well tolerated by primary neurons and hepatocytes [19]. To evaluate further the potential therapeutic application of this compound, we assessed whether it is also well tolerated after in vivo administration. Triflorcas was intra-peritoneally injected into mice at a dose of 30 mg.kg^-1^ each day. As body weight is a generic indicator of animal physiology influenced, for example, by metabolism, animal activity, and feeding behavior, the weight of Triflorcas-treated mice was followed over time. No significant differences were found versus controls and throughout treatment (*P*>0.05; Figure 3A). We also measured the weight of the heart, spleen, kidney, and liver of mice treated for 21 days and no differences were found between the two groups (*P*>0.05; Figure 3B).

We previously showed that Triflorcas elicits tumor growth inhibition of GTL-16 cells in vivo (Figure S2) [19]. We therefore determined whether the antitumor action of Triflorcas observed in vitro on H1437 cells might also be evidenced in vivo using xenografted nude mice. H1437 cells (5×10^6^) were sub-cutaneously injected into nude mice. After tumor formation, the mice were treated with Triflorcas, crizotinib, or vehicle alone, and tumor growth was examined during and after treatment. Notably, we found a 58.7% and 59% reduction in tumor volume when Triflorcas was administered at a dose of 30 mg.kg^−1^ or 60 mg.kg^−1^ every other day, respectively (control: 82.4 mm^3^±78.2; 30 mg.kg^−1^ Triflorcas injection: 34.0±24.5; *P* = 0.04; 60 mg.kg^−1^ Triflorcas injection: 33.8±24.2; *P* = 0.04; Figure 3C). A reduction of tumor weight was also observed in Triflorcas-treated mice (control: 147.1 mg ±92.5; 30 mg.kg^−1^ Triflorcas injection: 79.1±58.1, *P* = 0.03; 60 mg.kg^−1^ Triflorcas injection: 62.0±38.9, *P* = 0.01; Figure 3D). In contrast, no reduction in tumor growth was found in mice treated with crizotinib at doses of 50 mg.kg^−1^ every day (tumor volume: 118.6 mm^3^±69.4; tumor size: 205.0 mg ±84.8; Figure 3C and D), consistent with previous studies [21]. Taken together, these findings demonstrate that in vivo Triflorcas elicits tumor growth inhibition of cancer cells with oncogenic Met. Moreover, the absence of side effects indicates that Triflorcas is well tolerated when injected into mice at doses required to elicit its anti-tumor effects.

### Minor Changes in Gene Expression Profile of Stress and Toxicity Pathways by Triflorcas Correlate with Lack of Toxic Effects in vivo

**Figure 4:**
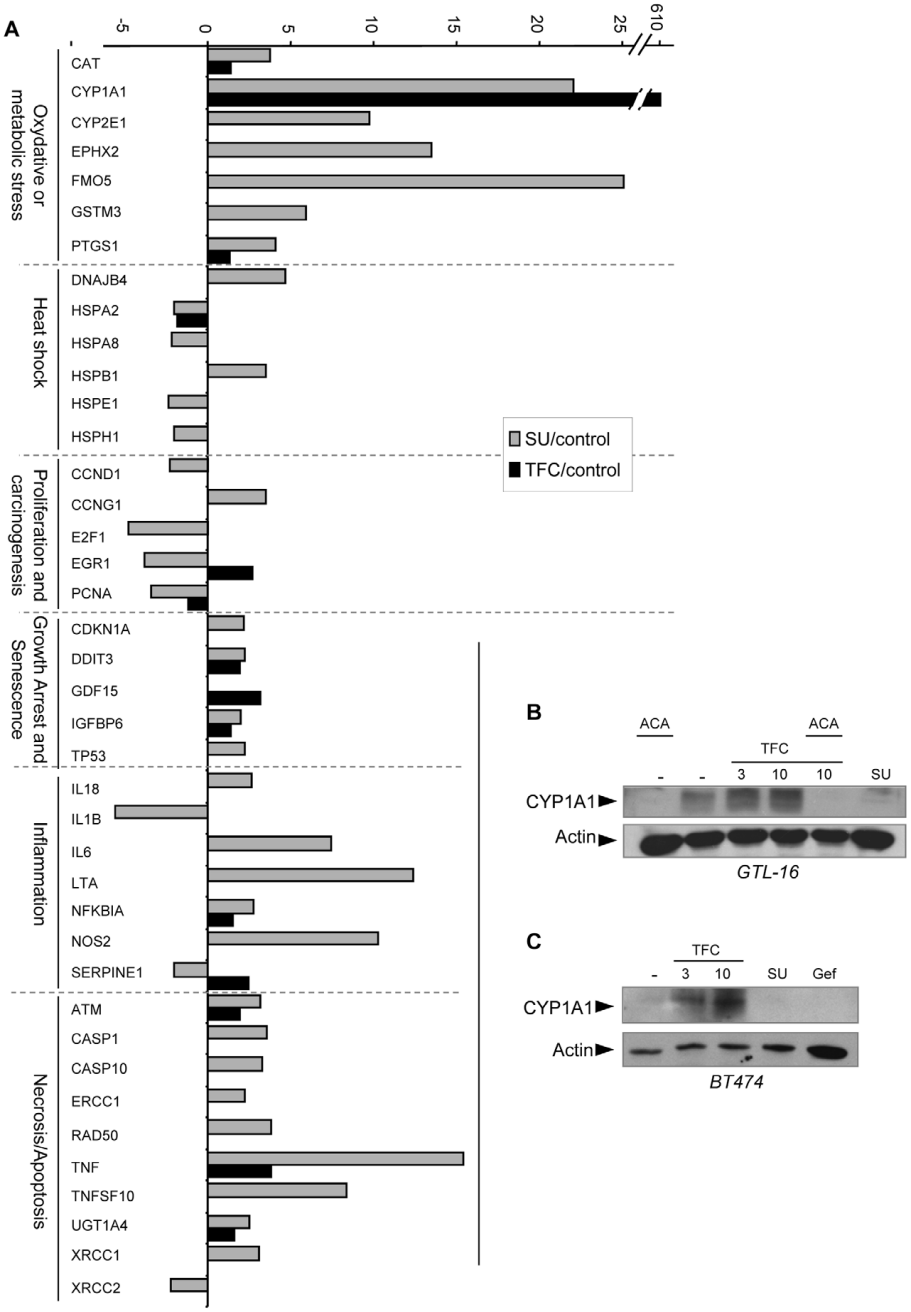
Triflorcas elicits a selective gene expression profile on stress and toxicity pathways. (A) The expression profile of 84 genes related to cell stress and toxicity was analyzed in GTL-16 cells. Cells were treated with either Triflorcas (black columns; 3 µM) or SU11274 (grey columns; 1 µM) for 24 hours, and gene expression was compared to that of untreated cells. Genes were grouped in clusters corresponding to oxidative/metabolic stress, heat shock, proliferation/carcinogenesis, growth arrest/senescence, inflammation, and apoptosis/necrosis signaling. Only statistically significant changes in gene expression are indicated (*P*<0.05). Triflorcas altered the expression of only 14 genes compared to the alteration of 39 genes induced by SU11274 treatment. Notably, the expression of *cyp1A1* was increased 611-fold in the presence of Triflorcas. (B) Western blot analysis showing the up-regulation of CYP1A1 protein levels in cells exposed to Triflorcas (3 or 10 µM). Acacetin (ACA; 10 µM) treatment prevented CYP1A1 up-regulation by Triflorcas (TFC). (C) CYP1A1 up-regulation by Triflorcas also occurred in ErbB1-addicted cancer BT474 cells. Gefitinib (Gef; 10 µM) and SU11274 (SU; 2 µM) were used as controls.

As Triflorcas is well tolerated both in vitro [19] and in vivo (Figure 3A and B), we next followed gene expression levels of a human RT-PCR array focused on toxicity and stress pathways. The expression profile of 84 genes related to cell stress and toxicity was analyzed in GTL-16 cells exposed to Triflorcas, SU11274, or vehicle. SU11274 altered the expression of 39 genes for at least 2-fold (by increasing or decreasing them; *P*<0.05). These genes belong to the apoptosis/necrosis (n = 10), inflammation (n = 7), oxidative/metabolic stress (n = 7), heat shock (n = 6), proliferation/carcinogenesis (n = 5), and growth arrest/senescence pathways (n = 4; Figure 4A and Table S1). In contrast, Triflorcas led to a statistically significant change in expression of only 14 genes (*P*<0.05). Among them, 13 genes overlapped with those altered by SU11274, and belonged to apoptosis/necrosis (n = 3), oxidative/metabolic stress (n = 3), and growth arrest/senescence (n = 3; Figure 4A and Table S1). No significant decrease of gene expression beyond 50% compared to control cells could be observed. Genes showing over a 2-fold increase in expression levels included *tnf* (3.83-fold; *P* = 0.0008), *gdf15* (3.18-fold; *P* = 0.0007), *egr1* (2.68-fold; *P* = 0.003), and *serpine1* (2.42-fold; *P* = 0.005). Intriguingly, Triflorcas led to a robust and predominant up-regulation of the cytochrome oxidase *cyp1a1* gene (611-fold; *P* = 0.0001; Figure 4A). Expression of the *cyp1a1* gene was also increased by SU11274, but at significant lower levels (22-fold; *P* = 0.02; Figure 4A). CYP1A1 is a member of the CYP1 family of cytochrome P450 implicated in cancer cell response to therapeutic agents. Western blot analysis confirmed the up-regulation of CYP1A1 protein by Triflorcas, which was impaired by its inhibitor acacetin (Figure 4B) [23]. CYP1A1 up-regulation by Triflorcas was independent of its action on Met as found also in cells, such as in the human breast cancer BT474 cells (Figure 4C), which are addicted to ErbB1 signaling. Together, these studies underline a selective metabolism activity profile elicited by Triflorcas linked to the up-regulation of a small subset of stress and toxicity genes. Thus, the minimal number of genes affected by Triflorcas indicates that this compound elicits more selective action on stress and toxicity genes compared to other Met anticancer agents such as SU11274.

### Triflorcas Mechanisms of Drug Action and its Effects on Cell Cycle Progression Leading to Mitotic Failure

**Figure 5:**
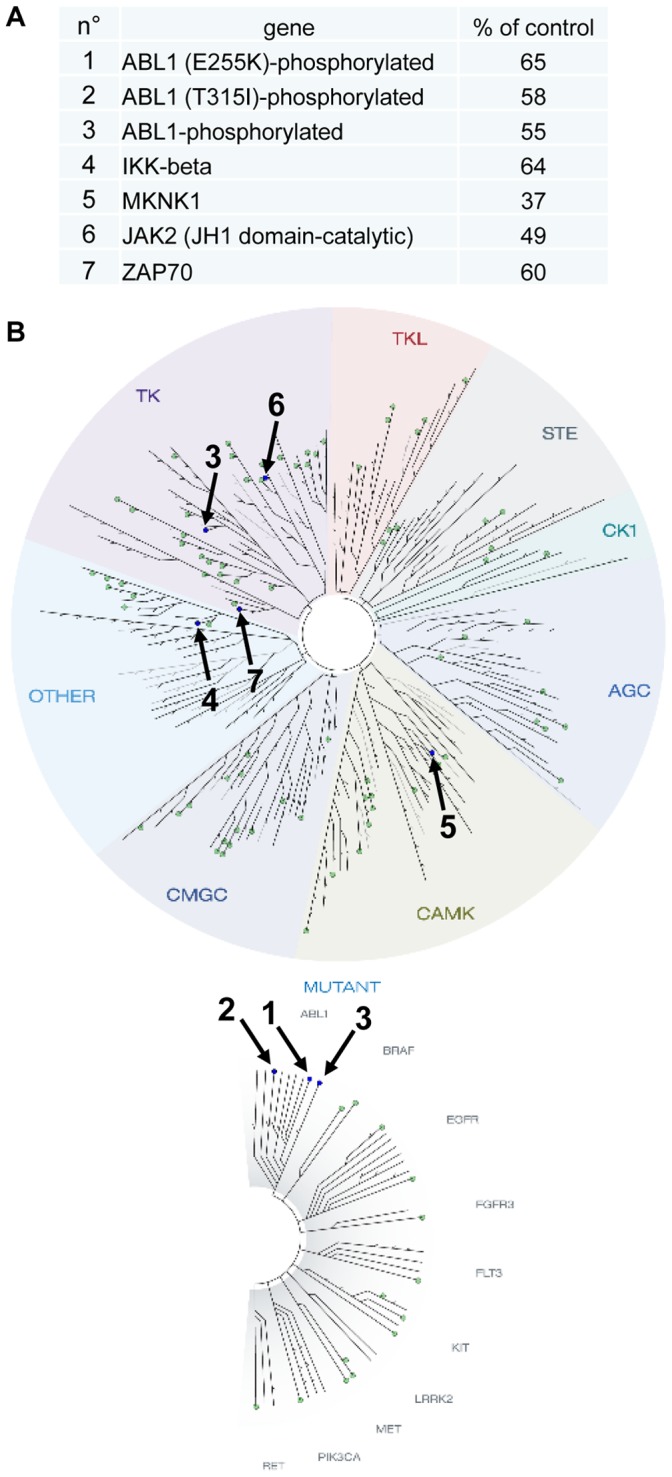
Small molecule kinase interaction map for Triflorcas. Compound was screened against a KINOME*scan* (http://www.kinomescan.com) panel of 98 kinases. (A) The table indicates the kinases for which Triflorcas reduced more than 30% the binding constant. The ligand binding for each kinase (% of control condition) is indicated. (B) TREEspot image of the 98 kinases screened with the position of Triflorcas targets: Abl wild-type, Abl E255K and Abl T315I mutant forms, IKK-beta, JAK2, MKNK1, and ZAP70. The complete dataset is shown in Table S2. TK: tyrosine kinase; TKL: tyrosine kinase like; STE: STE kinase; CK1: cell kinase1; AGC: PKA, PKC, PKG kinases; CAMK: calcium calmodulin-regulated kinase; CMGC: CDK, MAP, GSK, CDK-like kinase.

The effects of Triflorcas on the PI3K-Akt pathway evidenced by biochemical studies (Figure 2B and C) suggest that Triflorcas anticancer properties may be associated with its activity on distinct signaling targets in addition to Met. One screening approach broadly used to identify targets of a given chemical agent is the KINOME*scan*, which allows assessing the activity of compounds against a panel of kinases through binding assays [24]. Triflorcas was screened against 98 kinases at a single concentration of 10 µM, in agreement with standard protocols. However, the low solubility of Triflorcas in buffer conditions used for this type of screen limited the possibility of identifying targeted kinases. Nevertheless, we found that Triflorcas reduces by more than 30% the binding constant of only 5 over 98 kinases analyzed: Abl-1 (either wild-type, or E255K and T315I mutant forms), IKK-beta, JAK2, MKNK1, and ZAP70 (Figure 5 and Table S2). Although the pattern of kinases interacting with Triflorcas appears highly focused, these results must take into account that a proportion of targets were possibly not detected due to the low solubility of Triflorcas in buffer conditions used for this screen. For example, Met was not identified despite the fact that Triflorcas inhibitory activity was previously established using the Kinexus compound profiling service [19].

**Figure 6:**
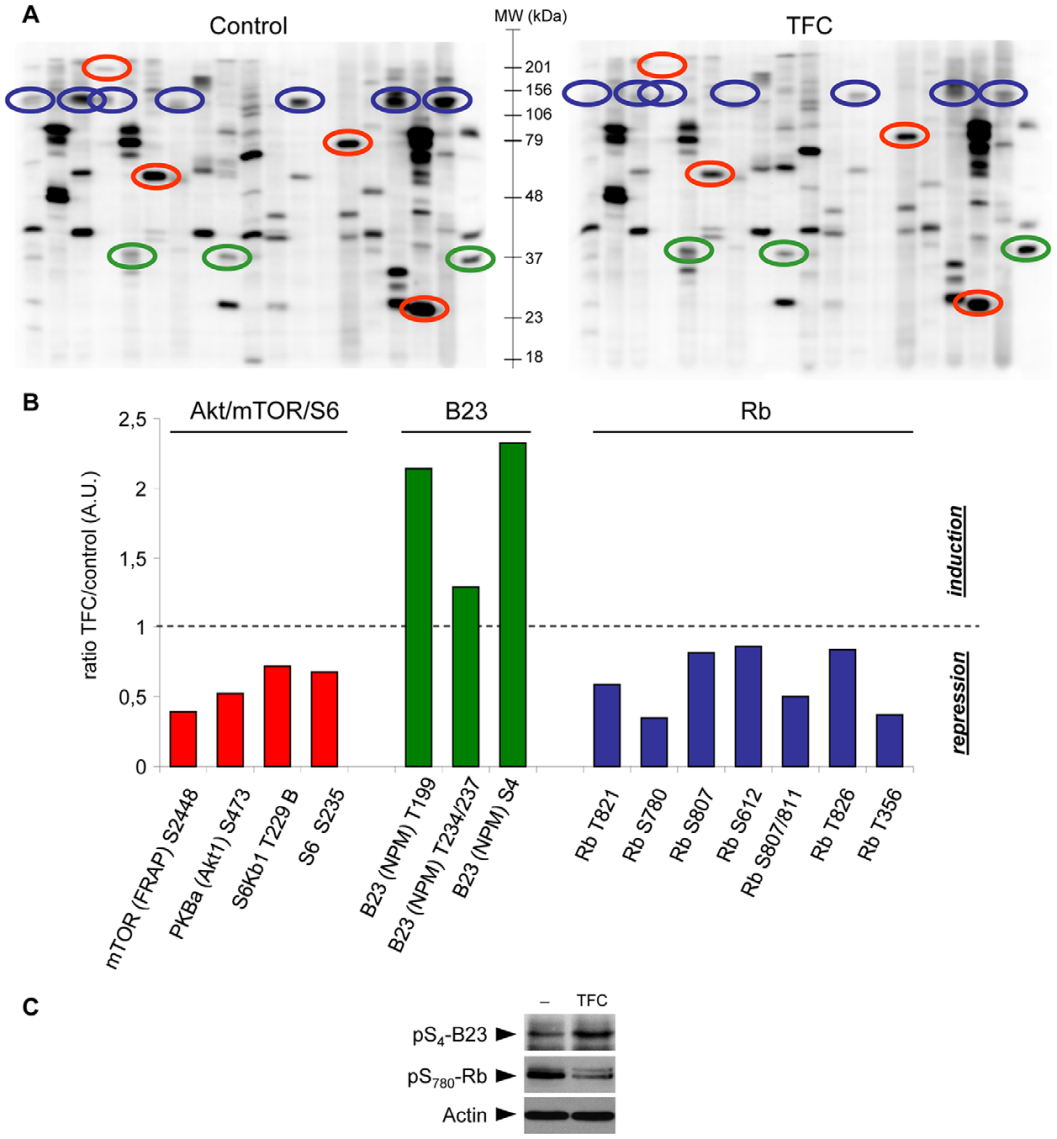
Triflorcas alters the phosphorylation status of cell cycle-related proteins. (A) The phosphorylation status of several cell cycle proteins was analyzed by applying the phospho-array KPSS 10.1 (Kinexus Bioinformatics). GTL-16 cells were treated with vehicle (control) or Triflorcas (TFC; 3 µM) for 72 hours (left and right panels, respectively). Red circles highlight constituents of the Akt/mTOR/S6 pathway. Green and blue circles surround nucleophosmin/B23 and Rb phospho-epitopes, respectively. (B) The graph shows the ratio of phosphorylation levels of the indicated proteins in cells untreated versus those exposed to Triflorcas. (C) Phosphorylation levels of nucleophosmin/B23 at Ser_4_ and Rb at S_780_ were increased and decreased, respectively, in GTL-16 cells exposed to Triflorcas (TFC; 3 µM for 24 hours).

Therefore, to further investigate the mechanism of drug action of Triflorcas, we applied a cell-based assay, which allows evaluation of the agent’s effects on multiple oncogenic signaling pathways in culture conditions compatible with Triflorcas solubility and biological activity. The phosphorylation levels of several signaling molecules were therefore examined by using the Kinexus phosphorylated protein screen array. In particular, we compared the phosphorylation levels of 44 signaling phospho-epitopes in GTL-16 cells treated or not with Triflorcas (Figure 6, S3, and Table S3). Consistent with our biochemical studies, we found that the phosphorylation state of several components within the PI3K/Akt pathway was altered in cells exposed to Triflorcas. In particular, reduced phosphorylation levels were observed for Akt1, mTOR/FRAP, p70^S6Kb1^, and S6 ribosomal protein (Figure 6A and B; red circles). Intriguingly, we found that Triflorcas significantly alters the phosphorylation status of two additional proteins: the phosphorylation of Rb on different Ser/Thr residues was reduced (Figure 6A and B; blue circles); the phosphorylation of nucleophosmin/B23, a nucleolar protein found to be significantly abundant in tumors [25], was increased (Figure 6A and B; green circles). Western blot analysis confirmed changes in the phosphorylation state of Rb and nucleophosmin/B23 proteins after Triflorcas treatment (Figure 6C).

**Figure 7:**
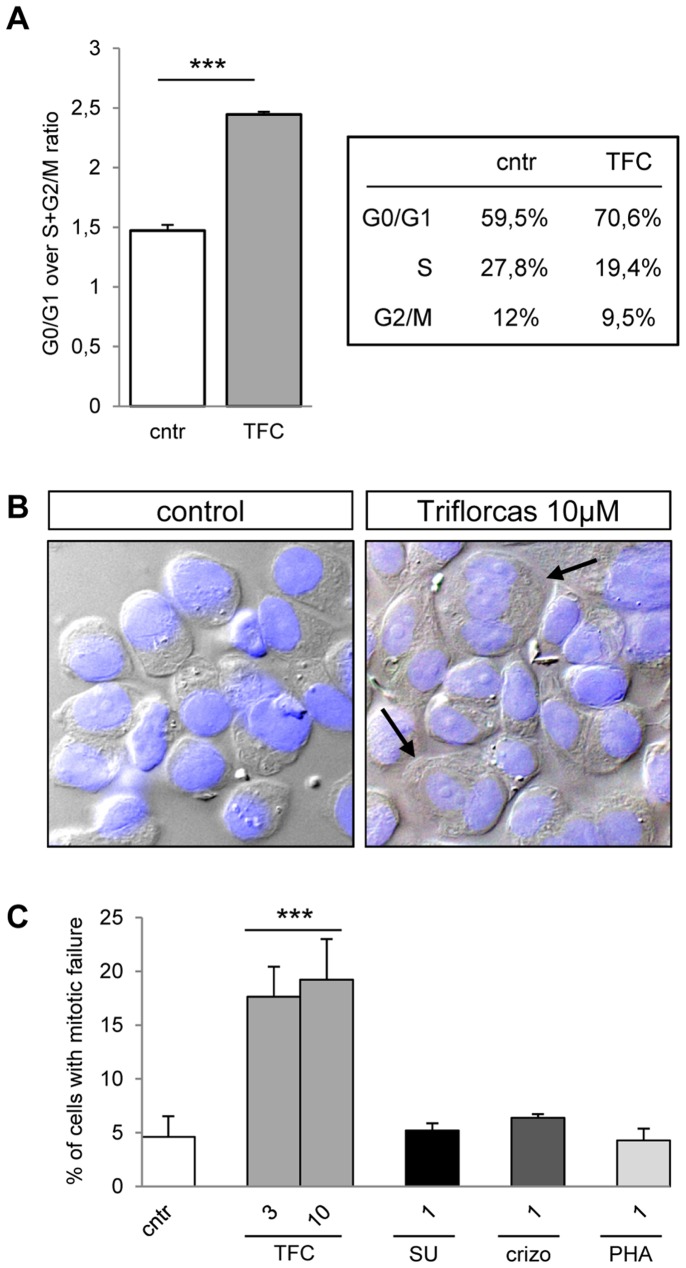
Triflorcas perturbs cell cycle progression, leading to mitotic failure. (A) Graph showing the ratio of percentage of GTL-16 cells in G0/G1 phase over the percentage of cells in S+G2/M phase. Cells were treated with Triflorcas (TFC; 3 µM), or vehicle (cntr) for 48 hours, then stained with propidium iodide. Values are expressed as means ± s.e.m. ***P<0.001; Student-*t* test (n = 3). Percentages of cells in G0/G1, S, and G2/M phases are reported in the table. (B) GTL-16 cells were grown in the presence of Triflorcas or vehicle and nuclei were visualized using DAPI staining. Arrows show example of cells with multiple nuclei (40X magnification). (C) Quantification of cells with mitotic failure expressed as percentage of the total number of cells analyzed. Triflorcas (TFC; 3 or 10 µM), but not SU11274 (SU; 1 µM), crizotinib (crizo; 1 µM), or PHA665752 (PHA; 1 µM) led to a significant increase in the number of cells with mitotic failure. Values are expressed as means ± s.e.m. ***P<0.001; Student-*t* test.

As both Rb and nucleophosmin/B23 are key regulators of cell cycle progression, we next evaluated whether these changes in phosphorylation state were paralleled with alteration of the cell cycle. Cells were stained with propidium iodide and their distribution in different cell cycle phases was assessed by flow cytometric analysis. Untreated GTL-16 cells were proliferating, as confirmed by the flow cytometry pattern (Figure 7A and data not shown). Triflorcas treatment alters GTL-16 cell cycle distribution, with an increase of the G0/G1 cell population at the expense of the S and G2/M population (Figure 7A and data not shown). As controls, treatment of GTL-16 cells with either SU11274 or nocodazol led to a blockage into G0/G1 or G2/M phase, respectively (data not shown). Thus, Triflorcas affects cell cycle progression of GTL-16 cells. Morphological analysis of cells exposed to Triflorcas showed a significant increase in the number of multinucleated cells, which indicates mitotic failure (Figure 7B and C). In contrast, no mitotic failure was observed in cells treated with SU11274, crizotinib, or PHA665752 (Figure 7C). Together, these findings show that the anti-tumor activity elicited by Triflorcas may in part account for phosphorylation changes of distinct signaling targets, such as components of the PI3K/Akt pathway, Rb, and nucleophosmin/B23, which correlates with alterations in cell cycle progression and mitotic failure.

### Triflorcas Impairs Survival and Anchorage-independent Growth of Human Cancer Cells Characterized by RTK Swapping

**Figure 8:**
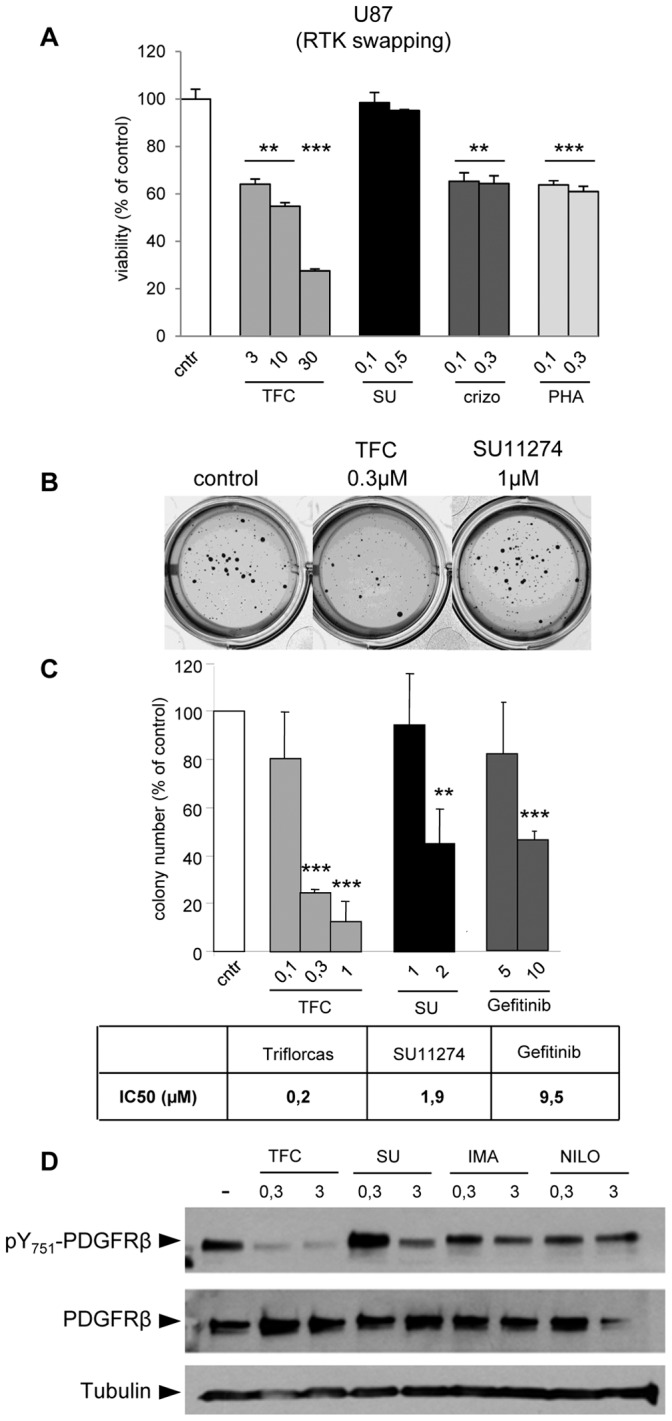
Triflorcas impairs survival and in vitro tumorigenesis of cancer cells resistant to single RTK inhibition through RTK swapping by also interfering with PDGFRβ phosphorylation. (A) Survival of U87 cells was reduced by Triflorcas in a dose dependent manner (µM; n = 2). SU11274 (SU), crizotinib (crizo), and PHA665752 (PHA) were used at the indicated concentrations (µM). (B and C) Triflorcas (TFC) impaired anchorage-independent growth of U87 cells, in a dose dependent manner (µM; n = 3). Gefitinib and SU11274 were used to inhibit ErbB1 and Met, respectively. (D) PDGFRβ phosphorylation in U87 cells was drastically reduced in presence of Triflorcas. For western blot analyses, cells were cultured in presence or absence of Triflorcas, SU11274 (SU), Imatinib (IMA), or Nilotinib (Nilo) (0.3–3 µM). Total cell lysates were analyzed using anti-phospho-Y_751_-PDGFRβ (pY_751_-PDGFRβ) (upper panel), anti-PDGFRβ (middle panel), or Tubulin (bottom panel). Values are expressed as means ± s.e.m. **P<0.01; ***P<0.001; Student-*t* test.

One major limitation of molecular therapies using agents targeting distinct RTKs is the drug resistance mechanism, which can be either constitutive or acquired after treatment. In this context, it has been shown that Met, ErbBs, and PDGFRs can reciprocally substitute for each other to maintain the activity of RTK-driven oncogenic pathways [9], [26]–[29]. We therefore evaluated the inhibitory properties of Triflorcas in cancer cell lines, in which reciprocal substitution of Met, ErbBs, and PDGFRβ confers resistance to single RTK inhibition. Human glioblastoma-astrocytoma U87 cells were used as a model of RTK swapping [27]. Survival and anchorage-independent growth assays were performed by comparing the effectiveness of Triflorcas to that of other Met inhibitors. We found that Triflorcas impaired U87 cell survival in a dose-dependent manner compared to SU11274, crizotinib, and PHA665752 (Figure 8A). Notably, Triflorcas drastically reduced anchorage-independent growth of U87 cells, whereas both SU11274 and Gefitinib (ErbB1 inhibitor) elicited only moderate anchorage-independent growth inhibition (Figure 8B and C), as previously shown [27]. By evaluating the compound IC_50_, we found that the Triflorcas inhibitory effects were elicited at lower doses compared to those required in GTL-16 Met-addicted cells (U87∶0.2 µM; GTL-16∶0.811 µM), and more effectively than those elicited by SU11274 or Gefitinib (1.9 µM and 9.5 µM, respectively; Figure 8C).

We therefore investigated whether Triflorcas acts also on other target(s) than Met. ErbB1 and PDGFRβ were two obvious candidates as they are responsible for RTK swapping. We excluded that Triflorcas acts on ErbB1 as: a) ErbB1 phosphorylation was unaffected by Triflorcas in BT474 cells (Figure S1C) [19], and b) ErbB1 is not predominantly phosphorylated in U87 cells under normal conditions (data not shown), as previously reported [27]. In contrast, we observed a drastic reduction in PDGFRβ phosphorylation when cells were exposed to Triflorcas (Figure 8D). Remarkably, Triflorcas efficiently reduced PDGFRβ phosphorylation at lower doses compared to Imatinib or Nilotinib (Figure 8D), two agents targeting PDGFRβ, c-Kit, and c-Abl [30]. Triflorcas almost abolished PDGFRβ phosphorylation in U87 cells at 0.3 µM, whereas Imatinib or Nilotinib partially reduced PDGFRβ phosphorylation only at 3 µM (Figure 8D). Together, these findings show that Triflorcas exerts its anti-tumorigenic activity also on cancer cells with oncogenic RTK swapping.

### Triflorcas Elicits a Distinct Growth Inhibitory-response Profile in Cancer Cell Lines

**Figure 9:**
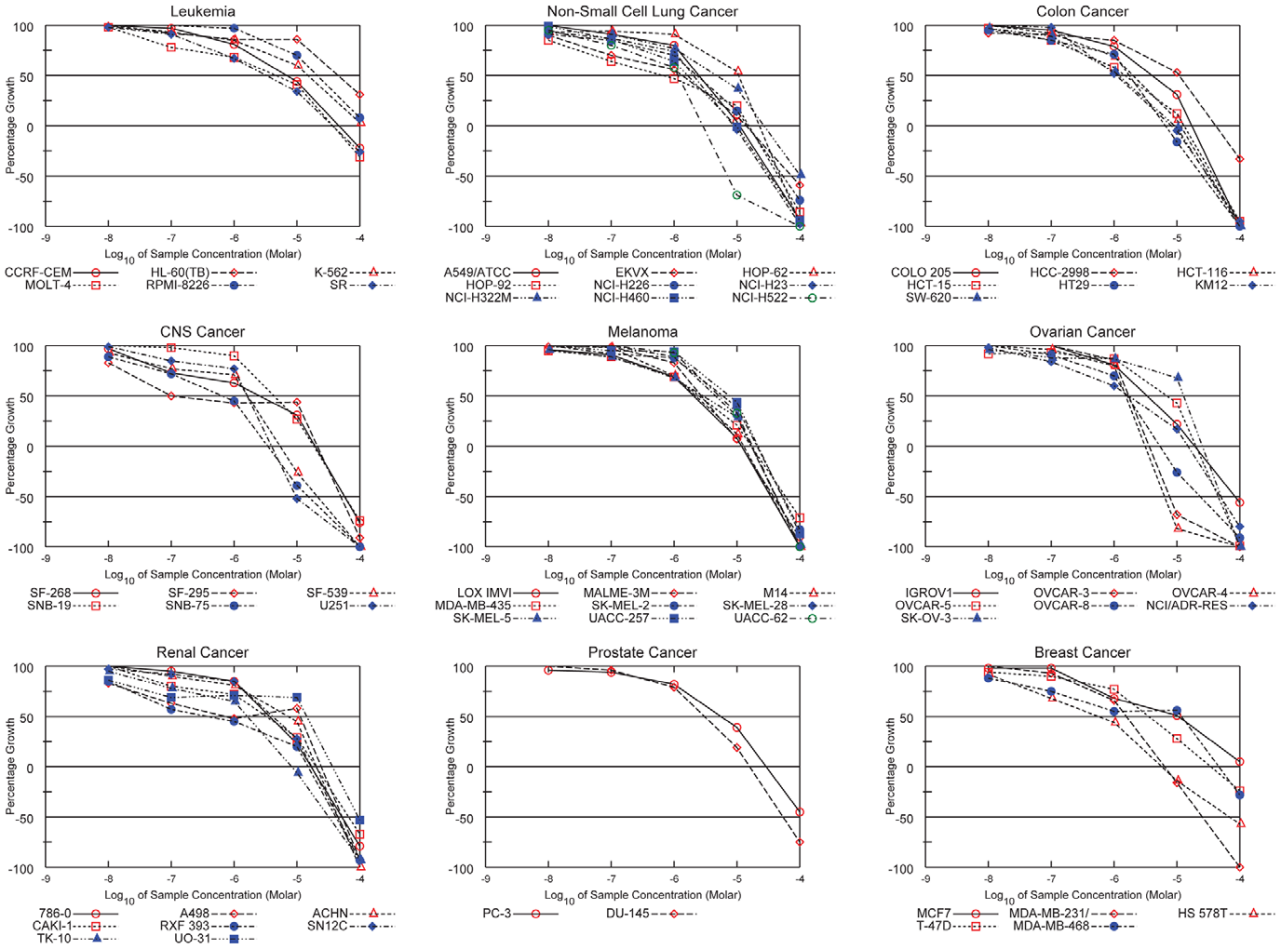
Cytotoxic effects of Triflorcas on the NCI60 panel of cancer cell lines. The cytotoxic effects of Triflorcas (10, 100 nM, 1, 10, 100 µM) was determined by sulphorhodamineB assay after 48 hours of drug treatment. Results shown are representative of two independent experiments.

To further elucidate the anticancer properties of imidazo[2,1-*b*]benzothiazol-2-ylphenyl moiety-based agents, we evaluated Triflorcas bioactivity by applying the disease-oriented NCI Anticancer Drug Screen [31]. This developmental therapeutic program historically allowed the efficient capture of compounds with anti-proliferative activity. In a preliminary test, Triflorcas was assayed at a single concentration of 10 µM in the full NCI60 cancer cell line panel (Figure S4). As it satisfied predetermined threshold inhibition criteria established for the NCI Anticancer Drug Screen, according to a minimum number of targeted cell lines, Triflorcas anticancer activity was then evaluated by using a full range of concentrations, in agreement with standard protocols (10, 100 nM, 1, 10, 100 µM). Results were expressed as the percentage of living cells following 48 hours of incubation (Figure 9). A decrease in cell number was seen in a proportion of cancer cells, with the mean log_10_ GI_50_ (growth inhibition) of –5.5±0.5M (Figure S5). The mean log_10_ LC_50_ (lethal concentration) calculated for these cell lines was –4.3±0.3M (Figure S5).

The COMPARE algorithm allows the identification of compounds whose pattern of growth inhibition is similar to the agent of interest [31]. Using this approach, we found that the Triflorcas activity correlated only minimally with that of known standard chemotherapeutic drugs (maximal correlation 0.335; Table S4). We further widened the comparison to the publicly available data from synthetic compounds screened on the NCI60 cancer cell line panel, and found that none of these compounds significantly matched with Triflorcas, as maximal correlation reached only 0.63 (Table S5). Thus, the unique growth inhibitory-response profile on cancer cells corresponding to solid tumors and leukemia indicates that imidazo[2,1-*b*]benzothiazol-2-ylphenyl moiety-based agents are characterized by a novel mechanism of drug action.

**Figure 10:**
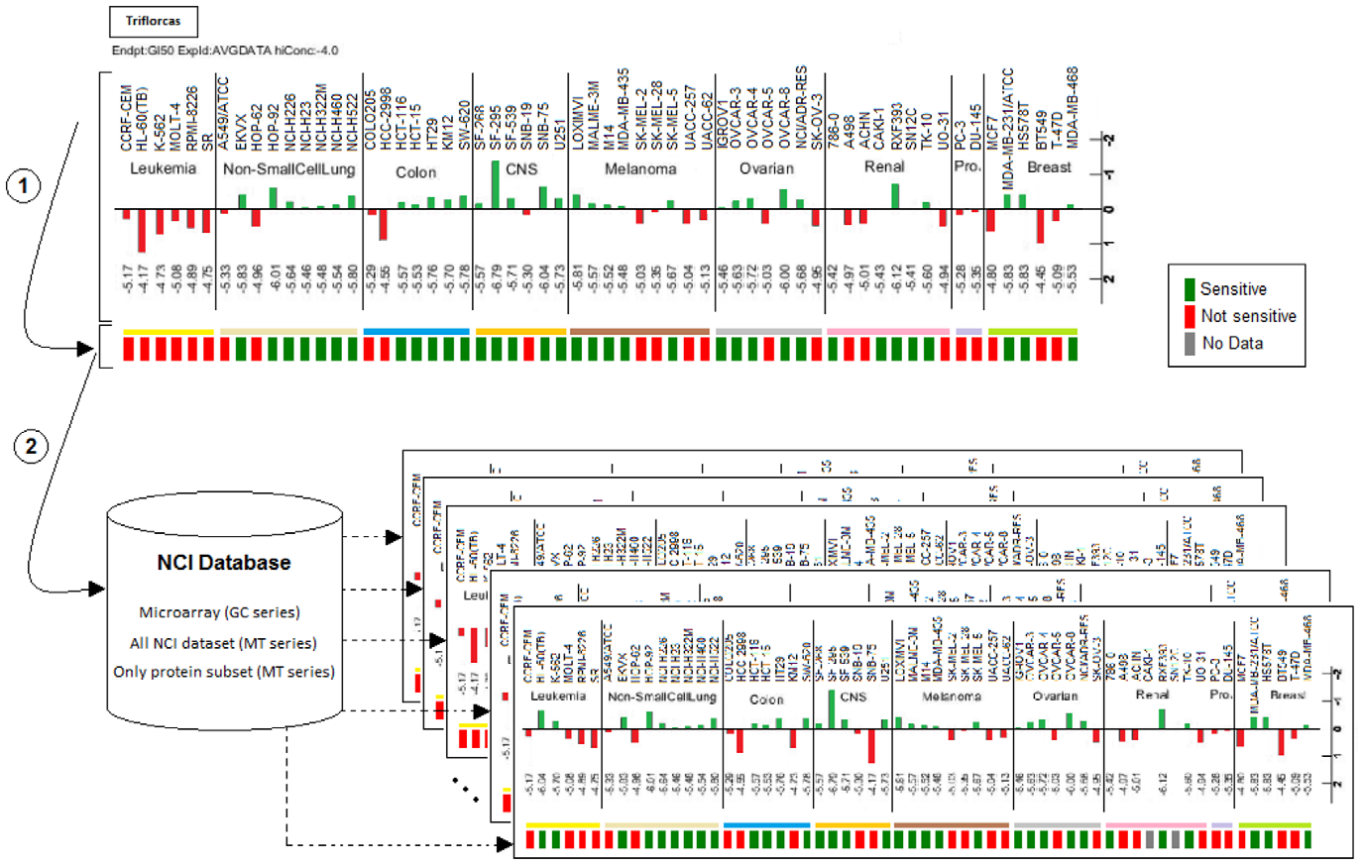
Schematic representation of bioinformatic methodology applied to the NCI Anticancer Drug Screen outcomes. The scheme displays the bioinformatics strategy applied to compare the sensitivity profile of NCI cancer cells with changes in molecular signatures from NCI data resources. 1: Sensitiveness of NCI cell lines to Triflorcas was extracted by using the mean GI_50_ into a numeric format and standard scores were visualized using green and red squares to indicate cells responding or not to Triflorcas, respectively. 2: NCI data resources applied to these studies (whole set of 87,545 measured targets) included: “Microarrays”, “all NCI dataset”, and “only Protein subset NCI” (see Material and Methods). The same color code was applied to indicate positive (green square) or negative (red square) changes of each molecular target analyzed.

**Figure 11:**
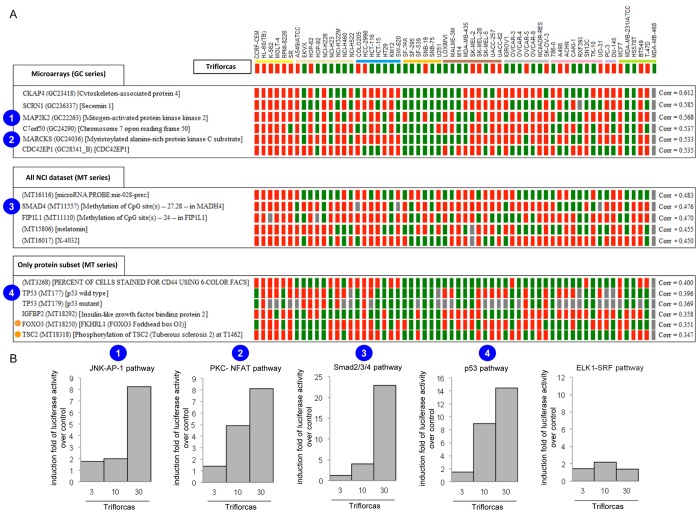
Molecular signature outcomes revealed by bioinformatic analysis of the NCI Anticancer Drug Screen. (A) Results showing a short list of molecular targets belonging to the individual datasets and displaying high positive correlation values (on the right). (B) GTL-16 cells were transfected with plasmids carrying the luciferase reporter gene controlled by the promoter of p53, Smad2/3/4, AP-1 (readout of the MAP2K2-JNK pathway), NFAT (readout of the PKC-MARCKS pathway), and Elk1-SRF (readout of ERK pathway). Luciferase activity was measured in cells after 48 hours of treatment with vehicle or Triflorcas (µM). Note that Triflorcas enhanced luciferase activity controlled by p53, Smad2/3/4, AP-1, NFAT, but not Elk1-SRF, promoters. Representative results of independent experiments are shown.

To get insights into potential molecular signatures characterizing cancer cells sensitive to Triflorcas, we performed bioinformatics studies using a large set of signaling database. In particular, we compared the response of the NCI60 cancer cell line panel to Triflorcas with NCI data resources from three databases: “Microarrays”, “All NCI dataset”, and “Only Protein subset NCI” (Figure 10). These studies highlighted a significant correlation between Triflorcas responsiveness and specific molecular changes (belonging to strong positive and weak positive correlation values). Signals with high correlation score included: cytoskeleton-associated protein4 (CKAP4), secernin1 (SCRN1), mitogen-activated protein kinase kinase 2 (MAP2K2), myristoylated alanine-rich protein kinase C substrate (MAPCKS), SMAD4, FIP1L1, p53, insulin-like growth factor binding protein 2 (IGFBP2), forkhead box O3 (FOXO3), and tuberous sclerosis 2 (TSC2) (Figure 11A). Notably, FOXO3 and TSC2 are known to be regulated by the PI3K/Akt pathway, which is targeted by Triflorcas (Figure 2 and 6). To further support bioinformatics outcomes, we experimentally assessed Triflorcas effects on signals highlighted in the three lists: “microarrays”, “all NCI dataset”, and “only protein subset NCI”. For this purpose, GTL-16 cells were transfected with luciferase reporter plasmids that enable measuring the activity of p53, Smad2/3/4, AP-1 (as read of the MAP2K2-JNK pathway) or NFAT (as readout of the PKC-MARCKS pathway) promoters. As Triflorcas does not affect ERK signaling (Figure 2B and C), an Elk1-SRF reporter plasmid was used as negative control. Luciferase activity was measured in cells after 48 hours treatment with vehicle or Triflorcas. Consistently with bioinformatics studies, we found that Triflorcas enhanced luciferase activity controlled by p53, Smad2/3/4, AP-1, NFAT, but not Elk1-SRF, promoters (Figure 11B). As JNK/AP-1 pathway activation can lead to distinct biological outcomes ranging from apoptosis induction to enhanced survival, tumor progression, and metastasis, according to its strength of stimulation and the signaling context [32], it will be relevant to assess JNK-AP-1 function on a panel of cancer cells sensitive to Triflorcas. Together, these studies show that compounds characterized by the imidazo[2,1-*b*]benzothiazol-2-ylphenyl moiety define a new class of chemical agents displaying anticancer activity towards distinct cancer cell types, according to molecular signatures indicated by bioinformatics studies.

## Discussion

Aberrant Met signaling in tumors recapitulates all the biological events controlled by Met during embryogenesis [33]–[42] and regenerative processes [43], [44]. To target oncogenic Met signaling, we originally generated a virtual chemical library of known anticancer agents and assessed their ability to interact with Met active site through computer modeling studies [12]. We reasoned that the flexibility of Met active site may offer the benefit of generating compounds in which anticancer properties and Met inhibitory features can be merged. The previously described imidazo[2,1-*b*]benzothiazol-2-ylphenyl compounds interact with Met active sites, as evaluated by in silico studies, interfere with Met phosphorylation, as assessed through biochemical and in vitro kinase assays, and hamper survival and anchorage-independent growth of Met-dependent cancer cells [19]. In the present study, we assessed the anticancer properties of Triflorcas, one of the most biologically active agents containing the imidazo[2,1-*b*]benzothiazol-2-ylphenyl moiety, on a panel of cancer cells. Our findings suggest that Triflorcas and its derivatives are promising agents to further exploit for targeting cancer cells: a) carrying Met amplification (such as GTL-16 cells, as previously shown [19]); b) carrying Met mutations (such as H2122 and H1437 cells); c) characterized by RTK swapping (such as U87 cells). By investigating the mechanism of drug action, we found that extinction of Met oncogenic signaling by Triflorcas occurs through at least three distinct mechanisms: a) by restraining Met activity [19], its phosphorylation, and phosphorylation of its immediate downstream signals such as Gab1; b) by enhancing Met internalization and degradation; c) by decreasing the phosphorylation levels of Akt and of its downstream targets mTOR, p70^S6K^, and S6 ribosomal protein, one pathway known to ensure Met dependency of cancer cells [22]. It is possibly the combination of these three actions that allows Triflorcas to be an effective inhibitor of cancer cells with oncogenic Met. Recent studies have highlighted the importance of intracellular trafficking to the cellular response of activated Met in tumorigenesis [45], [46]. As Triflorcas enhances Met internalization and degradation, it will be relevant to assess its properties on cancer cells carrying oncogenic forms of Met that render the receptor refractory to degradation [46]. We also show that the inhibitory properties of Triflorcas in cancer cells with RTK swapping can be partially attributed to its capacity to interfere with PDGFRβ phosphorylation.

Concerning the PI3K/Akt pathway, it is tempting to speculate that Triflorcas reduces its activation to a threshold level that becomes non-permissive for cancer cells when combined with inhibition of other oncogenic signals. Importantly, the reduction of PI3K/Akt pathway activation by Triflorcas, rather than its complete inhibition with more potent and selective drugs, might have the beneficial effect of minimizing side effects that limit the use of these latter in clinics [47], [48]. The effects on the PI3K pathway appear to be a direct action of Triflorcas on this pathway rather than being merely a consequence of its effects on Met. Several data support this hypothesis: a) selective inhibition of Akt function compromises in vitro tumorigenesis of H1437 and GTL-16 cells; b) decreased phosphorylation levels of the Akt, but not Ras/ERKs, pathway was observed in H1437 and GTL-16 cells; c) reduced Akt phosphorylation was observed also in ErbB1-addicted cells, which are resistant to Triflorcas. Beside its effects on the PI3K/Akt pathway, Triflorcas also influences the phosphorylation states of Rb and nucleophosmin/B23, two key regulators of cell cycle progression. Consistently, we found that Triflorcas treatment increases the G0/G1 cell population, leading to mitotic failure. Future studies will clarify how Triflorcas and its derivatives influence Rb and nucleophosmin/B23, whether there is a correlation between changes in their phosphorylation levels and alteration of the PI3K/Akt pathway, and whether these signaling alterations cause microtubular network dynamic instability and impaired mitotic spindle formation.

Our stress and toxicity RT-PCR array studies evidenced two additional properties of 2-phenylimidazo[2,1-*b*]benzothiazole derivatives. First, in contrast to SU11274, Triflorcas changes the expression of only 14 out of 84 genes, which are predominantly related to oxidative/metabolic stress, necrosis/apoptosis, and growth arrest/senescence. This limited alteration of stress and toxicity gene expression by Triflorcas correlates with the absence of major side effects observed in cultured neurons and hepatocytes [19], as well as in vivo (Figure 3A and B). Second, the expression levels of CYP1A1 are drastically up-regulated in tumor cells treated with Triflorcas. CYP1A1 belongs to the CYP1 cytochrome P450 family and has been implicated in cancer cell response to therapeutic agents by biotransforming them from prodrugs to active drugs [49]. CYP1A1 is the most up-regulated gene in cancer cells exposed to the benzothiazole derivative Phortress, its precursor (5F-203) or its desfluoro derivative (DF-203), chemotherapeutic prodrugs currently evaluated in clinical trials [50]. It is well established that these benzothiazole derivatives up-regulate, bind covalently to, and are metabolically bioactivated by CYP1A1 in sensitive cells [51], [52]. As Phortress and Triflorcas contain a similar heterocyclic moiety (Figure S6), it is reasonable that this part of the molecule plays a relevant role in up-regulating CYP1A1 expression in cancer cells. Future studies will clarify whether CYP1A1 influences the effects of Triflorcas by generating specific metabolites. Comparing the profiles of NCI cancer cells responding to Triflorcas and to benzothiazole compounds [49], [51], we intriguingly found a limited overlap, indicating that Triflorcas hampers cancer cells with mechanisms of drug sensitivity distinct to Phortress and its derivatives. We have also evaluated the functional relevance of CYP1A1 up-regulation by Triflorcas for its anti-tumorigenic activity and found that CYP1A1 pharmacological impairment did not significantly influence the inhibitory properties of Triflorcas on cell survival and anchorage independent growth (data not shown). Although the up-regulation of CYP1A1 does not appear to be the main event by which Triflorcas elicits its anti-tumor effects, we cannot exclude that, in some neoplastic cells, the modulation of anticancer pharmaceuticals by CYP1A1 may prove to be an advantage above additional mechanisms of action of 2-phenylimidazo[2,1-*b*]benzothiazole derivatives.

Genome-wide profiling and protein-network-based studies have recently established two important aspects related to cancer complexity. First, tumor evolution is often characterized by the acquisition of mutations in a small number of “core pathways” [4], [5]. Therefore, agents targeting a core pathway at distinct levels could restrain its oncogenic contribution and possibly minimize resistance mechanism. Second, RTKs share several effectors that participate in the oncogenic process and in the drug response [9]. This opens the possibility of designing anticancer therapies targeting mandatory signals in addition to hitting RTK activity directly. The benefit of combining drugs acting at distinct oncogenic levels is a well-established principle of cancer therapy, supported by several experimental settings [10]. However, determining which combinations would maximize effectiveness among the limitless possibilities remains a major challenge. Our studies establish that the flexibility of Met to accept different inhibitor binding modes offers the possibility to develop drugs targeting oncogenic Met signaling dependency at different levels as an alternative strategy. Although these agents might be less potent in directly impairing Met function compared to others discovered through exhaustive co-crystallographic studies, they may offer the possibility of lowering the activation of oncogenic dependency at non-permissive threshold levels, minimizing redundant pathways, either originally present in the tumor cells or acquired following treatment. Similarly to Sorafenib and here reported outcomes for Triflorcas, it is conceivable that compounds containing the imidazo[2,1-*b*]benzothiazol-2-ylphenyl moiety may have the ability to influence the oncogenic program by exerting modulation of distinct targets, as also evidenced by the disease-oriented NCI Anticancer Drug Screen. Thus, its novel mechanism of drug action together with a favorable side effect profile makes the 2-phenylimidazo[2,1-*b*]benzothiazole a relevant moiety to be further explored for the treatment of a broad range of tumor types.

## Materials and Methods

### Cell Culture

Human non-small-cell lung cancer (NSCLC) H1437 and H2122 cell lines, human breast cancer BT474 cells, and human glioblastoma-astrocytoma U87 cells were acquired from the America Type Culture Collection. Human gastric carcinoma GTL-16 cells are subclones of MKN45 cells (from Riken Cell Bank) obtained by limiting dilution [22]. H1437, H2122, BT474, and GTL-16 cells were grown in RPMI medium (Gibco-BRL), whereas U87 cells in Eagle’s Minimum Essential Medium (ATCC). Culture media were supplemented with 4mM L-glutamine and supplemented with 10% (v/v) fetal bovine serum (Gibco-BRL), 100 U/mL penicillin, and 100 µg/mL streptomycin. Cells were kept at 37°C in a humidified atmosphere of 5% CO_2_. H2122 and H1437 cells were used for survival and anchorage independent growth assays, respectively, according to their suitability for these biological assays.

### Compound Treatments

SU11274, Gefitinib, LY294002, acacetin, andresveratrol were purchased from Calbiochem; chlorpromazine and nocodazol from Sigma; crizotinib (PF-2341066) from Active Biochemicals; PHA665752 from Tocris Bioscience; Imatinib and Nilotinib were kindly provided by E. Buchdunger and P. Manley (Novartis Pharma AG, Basel, Switzerland); A-443654 (Akt inhibitor) was kindly provided by V.L.Giranda (ABBOTT Laboratories, Illinois, USA). For survival assays (H2122, GTL-16, and BT474 cells) and cell cycle analyses (GTL-16 cells), cells were cultured in serum-free media for 24 hours prior to compound addition for 48 hours. Survival assays with U87 cells was carried out in 0.1% serum. Viability was assessed with the Cell-Titer-Glo-Luminescent-Assay (Promega). For in vitro tumorigenesis, soft agar growth assays were performed as previously described [19]. Data on biological assays are representative of three independent experiments performed in duplicate or triplicate.

### Biochemical and Immuno-cytochemical Evaluation of Met Inhibitors

Cells were treated with inhibitors for 24 or 72 hours, as reported in the corresponding figures. Total extracts were analyzed as described [37]. Antibodies used were anti-tubulin, anti-actin (Sigma), anti-phosphoY_1234–1235_-Met, anti-p70^S6K^, anti-phosphoT_389_-p70^S6K^, anti-phosphoT_202_Y_204_-p70^S6K^, anti-Akt, anti-phosphoS_473_-Akt, anti-S6, anti-phosphoS_235/236_-S6, anti-phosphoS_240/244_-S6, anti phosphoT_202_Y_204_-ERKs, anti-ERKs, anti- phosphoY_627_-Gab1, anti-phosphoY_751_-PDGFRβ, anti-PDGFRβ, and anti-phosphoY_1068_-ErbB1 (Cell Signaling), anti-ErbBs, anti-Met, and anti-CYP1A1 (Santa Cruz), anti-Gab1 (Upstate), Alexa 546-conjugated-goat anti-rabbit antibodies (Invitrogen).

### In vivo Assays

Mice were kept at the IBDML animal facilities. All procedures involving the use of animals were performed in accordance with the European Community Council Directive of 24 November 1986 on the protection of animals used for experimental purposes (86/609/EEC). The experimental protocols were carried out in compliance with institutional Ethical Committee guidelines for animal research (comité d’éthique pour l’expérimentation animale – Comité d’éthique de Marseille; agreement number D13-055-21 by the Direction départementale des services vétérinaires – Préfecture des Bouches du Rhône). To evaluate compound toxicity in vivo, the weight of mice treated with Triflorcas (intra-peritoneally (i.p.) injection: 30 mg.kg^−1^) or vehicle was measured before, during, and after treatment. For heart, spleen, kidney, and liver weight, mice were sacrificed after 21 days of daily Triflorcas or vehicle treatment. Tumor xenografts were established by sub-cutaneous injection of H1437 cells (5×10^6^) in nude mice (S/SOPF SWISS NU/NU; Charles River). Treatment was initiated when tumors achieved an average volume of 15mm^3^ (approximately 7 days after cell injection; n = 10 mice per group). Mice were injected with: Triflorcas (i.p. 30 or 60 mg.kg^−1^ of body weight) or vehicle every other day; crizotinib (oral gavage 50 mg.kg^−1^ of body weight, in agreement with standard protocol) daily. Triflorcas was formulated in Cremophor-EL:DMSO (1∶1, v/v) and diluted in sterile 0.9% (w/v) sodium chloride. This formulation is classically applied for the administration of different chemical agents and does not elicit toxic effects, as revealed by no changes in mouse body weight during treatment. Mice were then sacrificed after 21 days of treatment. Tumor volume was determined from caliper measurements of tumor length (L) and width (W) according to the formula LW^2^/2. Tumor size was measured every week and at the end of the experiment. Tumor weight was established at the end of treatment. Two independent assays were performed (n = 8 mice per group). For tumor xenograft studies with GTL-16 cells, cells (10^6^) were i.p. injected in nude mice. Mice were treated with Triflorcas (i.p. 30 mg.kg^−1^) or vehicle at day 1 and treatment was repeated every other day. Mice were then sacrificed after 21 days of treatment. Tumor nodules present in the peritoneal cavity were isolated and quantified according to their diameter and their total weight. Two independent assays were performed (n = 8 mice per group).

### Gene Expression Analysis

For gene expression analysis, GTL-16 cells were cultured in serum-free media for 24 hours at approximately 40% confluence, prior to compound addition for 24 hours (Triflorcas: 3 µM; SU11274∶1 µM). Total RNA was isolated from untreated or treated cells using the RNAeasy-Kit, processed with DNase (RNase-free DNase set), and purified on RNAeasy column (Qiagen). The quality of RNA was tested by using Picochip (Agilent Technologies). Gene profiling was done by the SuperArray Biosciences service using the RT2-profiler PCR array Stress and Toxicity Pathway Finder (96 genes). The data were imported into an Excel database and analyzed using the comparative cycle threshold method with normalization of the raw data to β-actin. The results are presented as *n*-fold changes versus the values of untreated cells. The mean value was calculated from measurements of three independent biological samples.

### KINOME*scan*

The activity of Triflorcas was assessed on a panel of 98 kinases through binding assays using the KINOME*scan* service. The vehicle alone was used as negative control. The KINOME*scan* is based on a competition binding assay that quantitatively measures the ability of a compound to compete with an immobilized active-site directed ligand.

### Kinexus

Cells were treated with Triflorcas (3 µM) or vehicle for 72 hours. Protein extraction was performed as described by the manufacturer (Kinexus Bioinformatics, Vancouver, Canada). Samples were then analyzed by KINEXUS service using the phospho-array KPSS-10.1.

### FACS Analysis

Cells were treated with Triflorcas (3 µM), SU11274 (1 µM), or vehicle for 48 hours. Cells were then fixed with 70% ethanol and washed twice in PBS before treatment with RNase 100 µg/mL. After staining with propidium iodide (50 µg/ml), cells were analyzed by flow cytometry.

### NCI60 Screening

Triflorcas was screened by the NCI towards a panel of 60 cell lines. Cell sensitivity was assessed and results were expressed as TGI (Total Growth Inhibition), GI_50_ (50% Growth Inhibition), and LC_50_ (50% Lethal Concentration). Triflorcas action was evaluated towards data from public resources including CellMiner and COMPARE software. The COMPARE algorithm was used to search for compounds tested on the NCI60 panel, to uncover similar sensitivity profile to the Triflorcas. Luciferase constructs (Cignal Reporters; QIAGEN) were used for reporter assay studies and experiments were performed according to the manufacturers instructions.

### Bioinformatics Studies

To perform bioinformatics studies, we used three datasets downloaded from the NCI data resources to identify potential molecular signatures of NCI cancer cells sensitive to Triflorcas (last update August 2010, http://dtp.nci.nih.gov/mtargets/download.html). These databases included: “Microarrays”, which contain 74,700 measured targets derived from large scale experiments (Affymetrix U133 from Chiron, Affymetrix U95A from Novartis, Affymetric HUM6000 from Millenium Pharmaceuticals, and cDNA array data from Weinstein (NCI) and Brown & Botstein (Stanford) groups); “all NCI dataset”, which contains 12,845 measured targets derived from all small-scale measurements such as protein, mRNA, miRNA, DNA methylation, mutations, SNPs, enzyme activity, metabolites, but excluding Microarray; “only Protein subset NCI”, which contains 333 measured targets derived from the NCI protein screening subset. The whole bioinformatics analysis involved three steps. First, to compare data, we used the developmental therapeutics program mean graph for the Triflorcas (five dose average data at GI_50_ endpoint) to extract the sensitivity level of each cancer cell line tested. We then extracted the expression level of signals scored in each NCI cancer cell line. To allow comparison between Triflorcas cell sensitivity values and molecular target expression values, each value was normalized according to a standard normal distribution (standard score defined as: mean = 0; standard deviation = 1). Second, we scored the correlation existing between Triflorcas sensitivity values with that of each molecular target value by using a statistical correlation coefficient algorithm. The correlation coefficient spans values between −1 and +1 and it permits to define five classes of correlation: strong negative correlation (−1 to −0.5), weak negative correlation (−0.5 to 0), weak positive correlation (0 to 0.5), strong positive correlation (0.5 to 1), and no correlation (0). Only positive correlations (weak or strong) were included in these studies in order to extrapolate mainly putative targets of Triflorcas. Third, results were displayed in order to verify the strength of the global (whole cell lines) or specific (individual cell line) correlation. For this purpose, we defined a specific color code to highlight differences and similarities between the sensitivity profile of Triflorcas and changes in molecular targets. The color code was assigned according to the standard score above described. All values above 0 represent sensitivity of a given cell line to Triflorcas and are indicated by green squares. All values below 0 stand for no sensitivity and are represented by red squares. The same approach was applied to encode the expression of a given molecular target in each cell line: values above 0 were represented by green squares; values below 0 were represented by red squares. As not all cell lines screened for Triflorcas were included into the analysis of some molecular targets, no available data were represented by gray squares and were not taken into account for statistical analysis. Finally, a web-based application was designed in order to: 1) automatically convert sensitivity of Triflorcas and changes in molecular targets into a numeric format compliant; 2) score the potential degree of correlation between cells response to Triflorcas and the large set of molecular targets, using a statistical correlation coefficient algorithm; 3) sort potential molecular targets according to their correlation score; 4) display the result in a web browser to retrieve a short list of molecular targets highlighting possible molecular signatures.

### Luciferase Reporter Assay

GTL-16 cells were transfected with luciferase reporter plasmids that enable measuring the activity of p53, Smad2/3/4, AP-1 (as read of the MAP2K2-JNK pathway) or NFAT (as readout of the PKC-MARCKS pathway), and Elk1-SRF (as read of ERK signaling) promoters (Cignal Reporters, Qiagen). Transfection was performed by using Lipofectamine according to the manufacturer's protocol (Invitrogen). After 24 hours of transfection, cells were treated or not with Triflorcas (3, 10, and 30 µM) for 48 hours, and then Luciferase activity was assessed. All Luciferase assays were performed using a dual Luciferase assay kit according to the manufacturer's protocol (Promega).

## Supporting Information

Figure S1 **Triflorcas acts on cancer cells carrying oncogenic Met, but not ErbB1.** (A) Anchorage-independent growth of H1437 cells was impaired in the presence of Triflorcas (TFC), LY294002 (PI3K inhibitor), or A443654 (Akt inhibitor). (B) Survival of GTL-16 cells was impaired in the presence of LY294002 (PI3K inhibitor) or A443654 (Akt inhibitor). Values are expressed as means ± s.e.m. **P<0.01; ***P<0.001; Student-*t* test. (C) Reduced phosphorylation levels of Akt, but not ErbB1, by Triflorcas was observed also in ErbB1-addicted BT474 cells. (D) BT474 cells were treated with Triflorcas (TFC), SU11274, or A443654 for 48 hours (µM), and viability was assessed with CellTiterGlo (control: cntr).
(TIF)

Figure S2 **Triflorcas interferes with tumor growth of Met-addicted cancer cells in nude mice.** Intra-peritoneal injection of GTL-16 cells in nude mice leads to the development of several nodules in the peritoneal cavity, offering the possibility to evaluate compound efficacy on tumor weight and nodule numbers. Triflorcas treatment (TFC; i.p. 30 mg.kg^-1^ every other day) reduced nodule numbers. Reduction of nodule numbers was of 82% for nodules smaller than 2 mm (vehicle: 8.6±4.6; TFC: 1.6±1.6; *P* = 4.4×10^−5^), 76% for nodules between 2 to 5 mm (vehicle: 10.6±5.3; TFC: 2.6±1.8; *P* = 5.2×10^−5^), and 59% for nodules bigger than 5 mm (vehicle: 2.3±1.6; TFC: 0.9±0.6; *P* = 9.9×10^−3^). Values are reported as boxplots and expressed as means ± s.e.m. **P<0.001; Student-*t* test.
(TIF)

Figure S3 **Triflorcas effects on the phosphorylation status of molecules regulating cell cycle.** Protein extracts from GTL-16 cells were analyzed for the phosphorylation status of different cell cycle proteins, using the Kinexus KPSS10.1 screen. Intensities are represented as normalized counts per minute. Blue and red lanes correspond to untreated or Triflorcas treated (3 µM) cells, respectively.
(TIF)

Figure S4 **Triflorcas effects on the NCI60 panel of cancer cell lines.** Triflorcas was applied to the NCI Anticancer Drug Screen, at a single concentration of 10 µM. Mean growth percentage is displayed. Mean graphs are constructed at each level of effect, with bars depicting the deviation of individual tumor cell line from the overall mean value for all the cells tested.
(TIF)

Figure S5 **Cancer cell sensitivity to Triflorcas.** Triflorcas was assessed on the NCI60 panel of cancer cells at different doses, ranging from 10 nM to 100 µM. Cell sensitivity is expressed as GI_50_ (50% Growth Inhibition), TGI (Total Growth Inhibition), and LC_50_ (50% Lethal Concentration).
(TIF)

Figure S6 **Chemical formulas of Triflorcas, Phortress, and other compounds characterized by the benzothiazole group.**
(TIF)

Table S1 **Ratios of gene expression analyzed with RT-PCR stress and toxicity array.** Cells were treated either with SU11274 (SU) or with Triflorcas (TFC). NS indicate not statistically significant changes.
(TIF)

Table S2 **Selectivity profile of Triflorcas.** Triflorcas was screened against a KINOME*scan* (http://www.kinomescan.com) panel of 98 kinases. The ratio of binding (% TCF over control condition) for each kinase is indicated. The arrows indicate the kinases for which Triflorcas reduced more than 30% the binding constant.
(TIF)

Table S3 **Triflorcas effects on the phosphorylation status of several cell cycle proteins analyzed by the phospho-array KPSS 10.1 (Kinexus Bioinformatics).** GTL-16 cells were treated with vehicle (CTRL) or Triflorcas (TFC; 3 µM) for 72 hours (first and second column, respectively). Intensities are represented as normalized counts per minute (CPM). Data for untreated or treated cells are reported. Changes in phosphorylation levels are expressed as either percentage or ratio of TFC-treated over control (third and fourth column, respectively).
(TIF)

Table S4 **Comparison of Triflorcas activity to standard agents in the NCI60 screen using the COMPARE software.** The 10 drugs most similar in Triflorcas activity are reported.
(TIF)

Table S5 **Comparison of Triflorcas activity to synthetic publicly available anticancer compounds in the NCI60 screen using the COMPARE software.** The 9 drugs most similar in activity are reported.
(TIF)
