# Supplementary figures and images for: Combined Drug Action of 2-Phenylimidazo[2,1-b]Benzothiazole Derivatives on Cancer Cells According to Their Oncogenic Molecular Signatures
Source: PLoS One. 2012 Oct 5;7(10):e46738. doi: 10.1371/journal.pone.0046738 (PMC3465283; doi:10.1371/journal.pone.0046738)

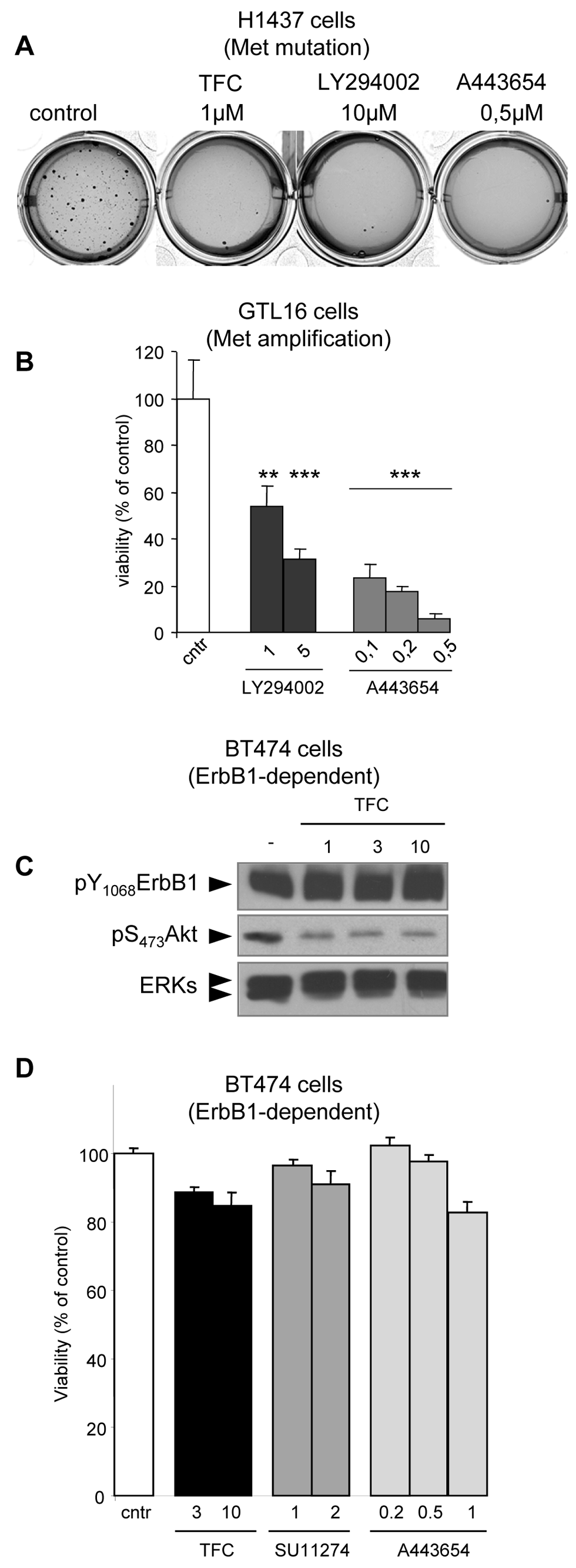

Supplement: Figure S1 — Triflorcas acts on cancer cells carrying oncogenic Met, but not ErbB1. (A) Anchorage-independent growth of H1437 cells was impaired in the presence of Triflorcas (TFC), LY294002 (PI3K inhibitor), or A443654 (Akt inhibitor). (B) Survival of GTL-16 cells was impaired in the presence of LY294002 (PI3K inhibitor) or A443654 (Akt inhibitor). Values are expressed as means ± s.e.m. **P<0.01; ***P<0.001; Student-t test. (C) Reduced phosphorylation levels of Akt, but not ErbB1, by Triflorcas was observed also in ErbB1-addicted BT474 cells. (D) BT474 cells were treated with Triflorcas (TFC), SU11274, or A443654 for 48 hours (µM), and viability was assessed with CellTiterGlo (control: cntr). (TIF) [file pone.0046738.s001.tif]

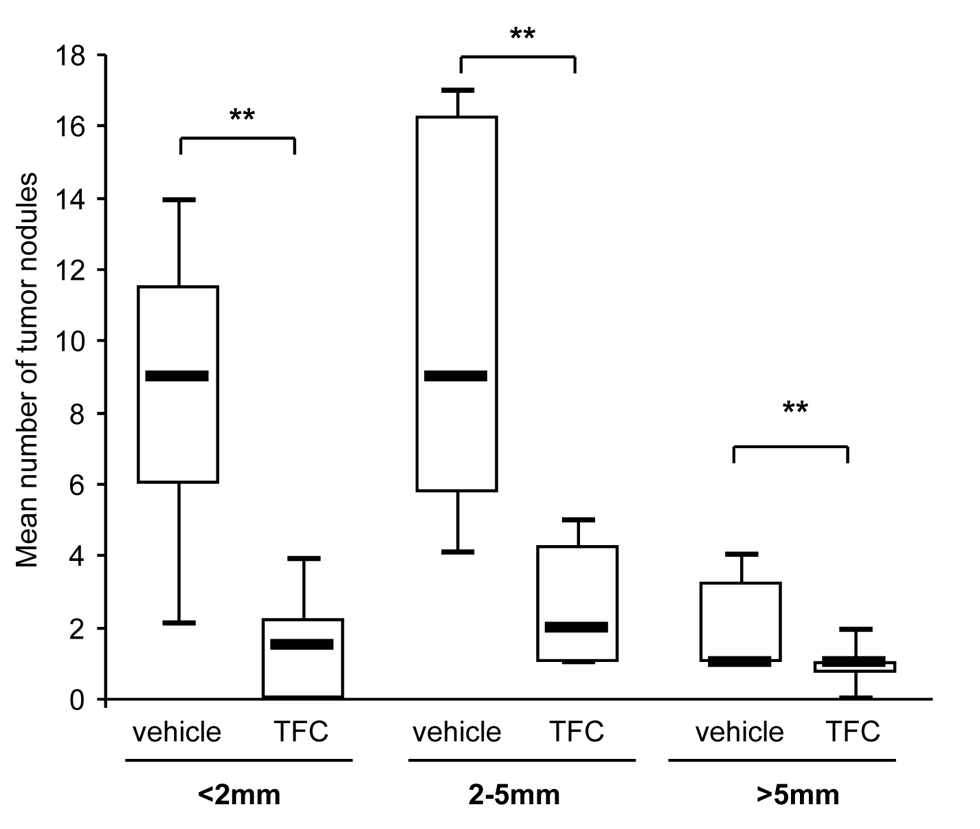

Supplement: Figure S2 — Triflorcas interferes with tumor growth of Met-addicted cancer cells in nude mice. Intra-peritoneal injection of GTL-16 cells in nude mice leads to the development of several nodules in the peritoneal cavity, offering the possibility to evaluate compound efficacy on tumor weight and nodule numbers. Triflorcas treatment (TFC; i.p. 30 mg.kg-1 every other day) reduced nodule numbers. Reduction of nodule numbers was of 82% for nodules smaller than 2 mm (vehicle: 8.6±4.6; TFC: 1.6±1.6; P = 4.4×10^−5^), 76% for nodules between 2 to 5 mm (vehicle: 10.6±5.3; TFC: 2.6±1.8; P = 5.2×10^−5^), and 59% for nodules bigger than 5 mm (vehicle: 2.3±1.6; TFC: 0.9±0.6; P = 9.9×10^−3^). Values are reported as boxplots and expressed as means ± s.e.m. **P<0.001; Student-t test. (TIF) [file pone.0046738.s002.tif]

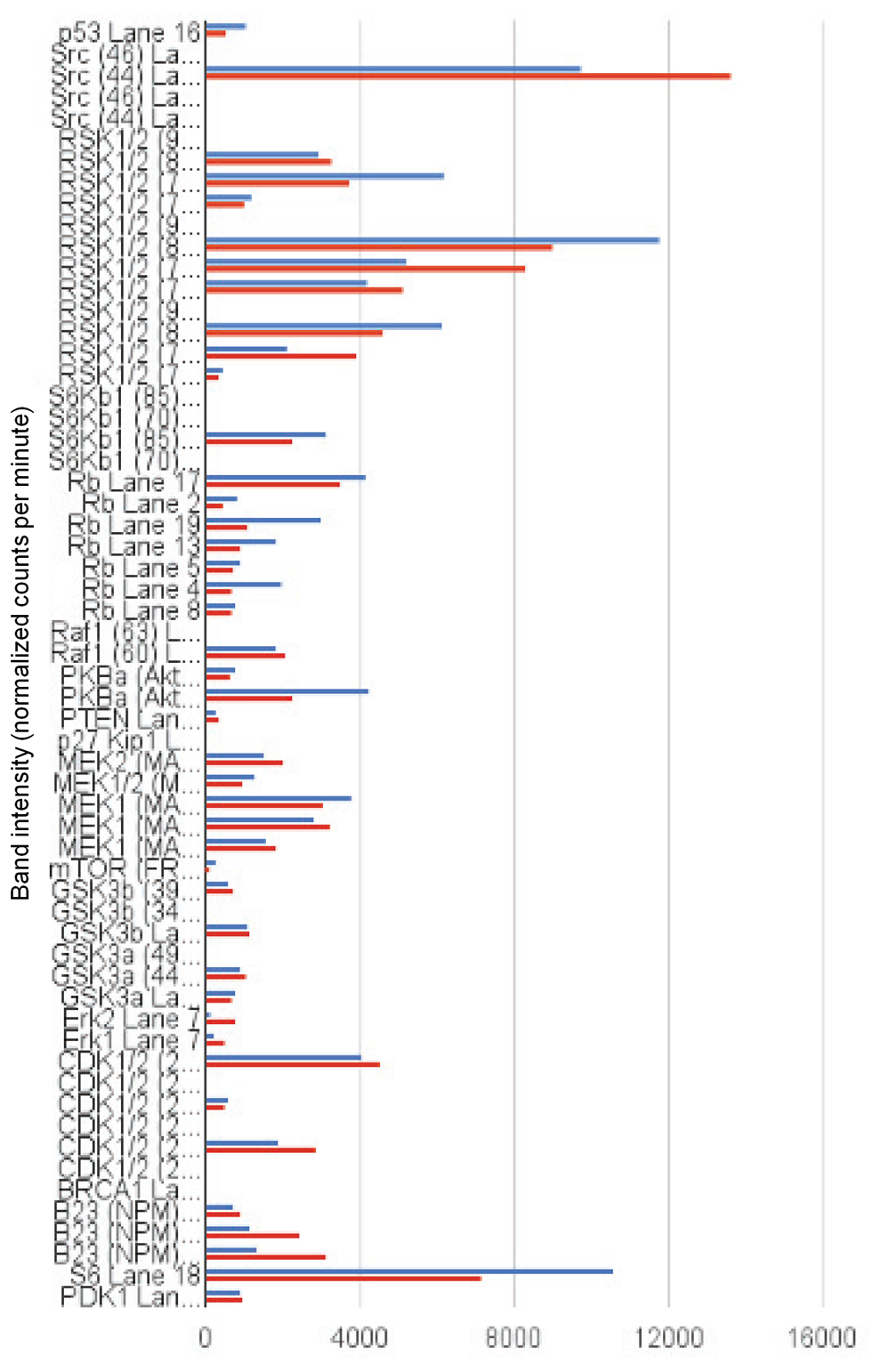

Supplement: Figure S3 — Triflorcas effects on the phosphorylation status of molecules regulating cell cycle. Protein extracts from GTL-16 cells were analyzed for the phosphorylation status of different cell cycle proteins, using the Kinexus KPSS10.1 screen. Intensities are represented as normalized counts per minute. Blue and red lanes correspond to untreated or Triflorcas treated (3 µM) cells, respectively. (TIF) [file pone.0046738.s003.tif]

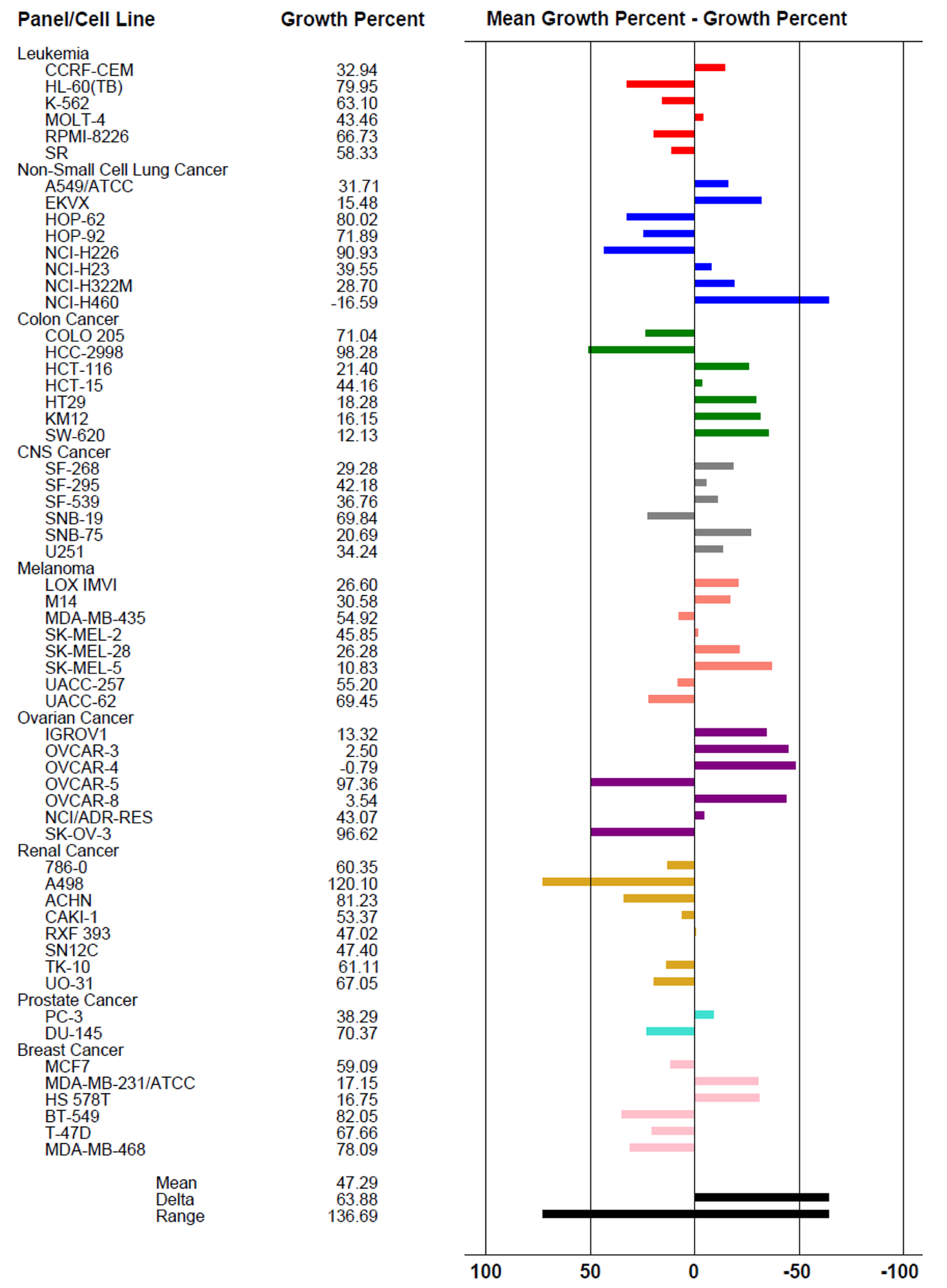

Supplement: Figure S4 — Triflorcas effects on the NCI60 panel of cancer cell lines. Triflorcas was applied to the NCI Anticancer Drug Screen, at a single concentration of 10 µM. Mean growth percentage is displayed. Mean graphs are constructed at each level of effect, with bars depicting the deviation of individual tumor cell line from the overall mean value for all the cells tested. (TIF) [file pone.0046738.s004.tif]

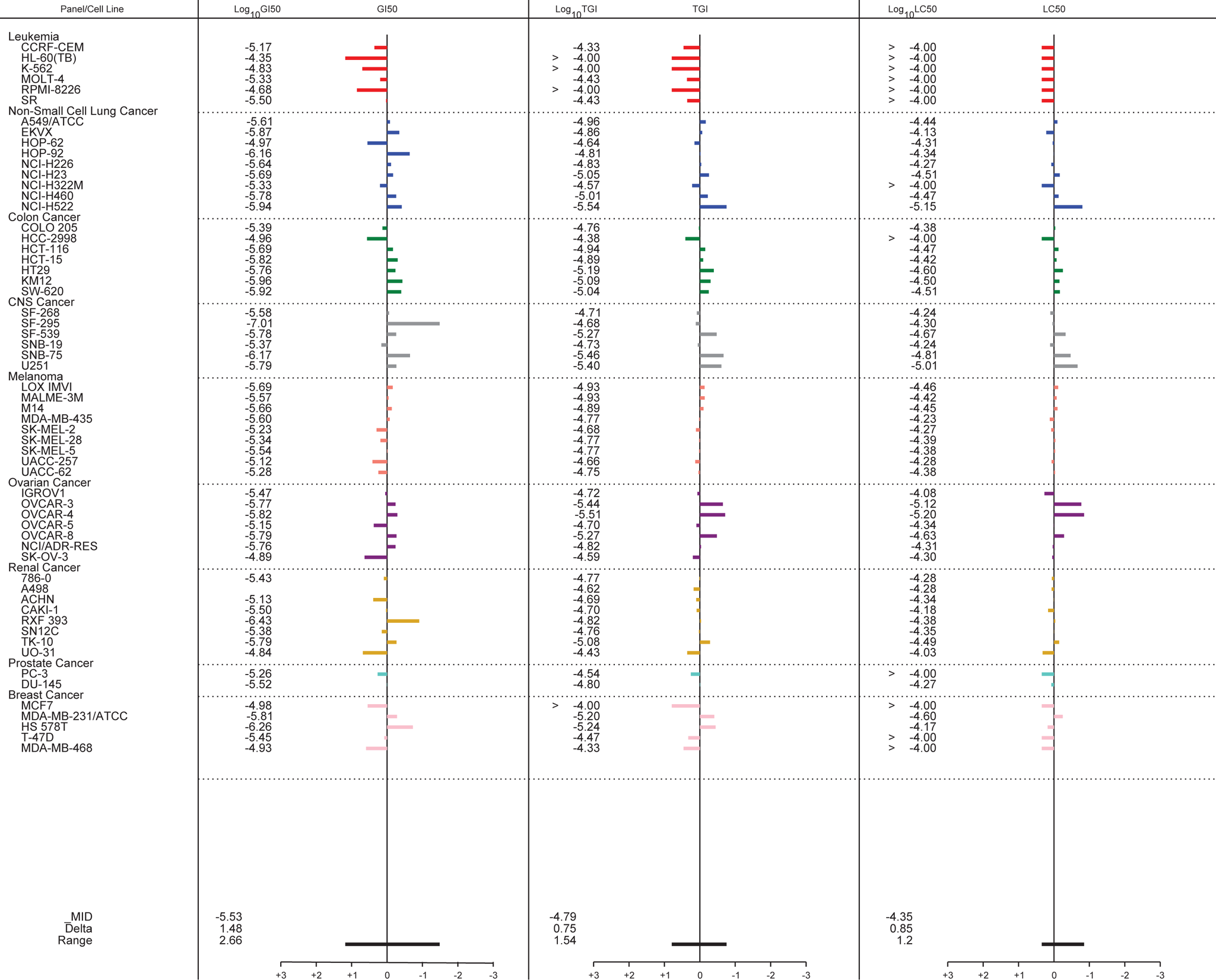

Supplement: Figure S5 — Cancer cell sensitivity to Triflorcas. Triflorcas was assessed on the NCI60 panel of cancer cells at different doses, ranging from 10 nM to 100 µM. Cell sensitivity is expressed as GI50 (50% Growth Inhibition), TGI (Total Growth Inhibition), and LC50 (50% Lethal Concentration). (TIF) [file pone.0046738.s005.tif]

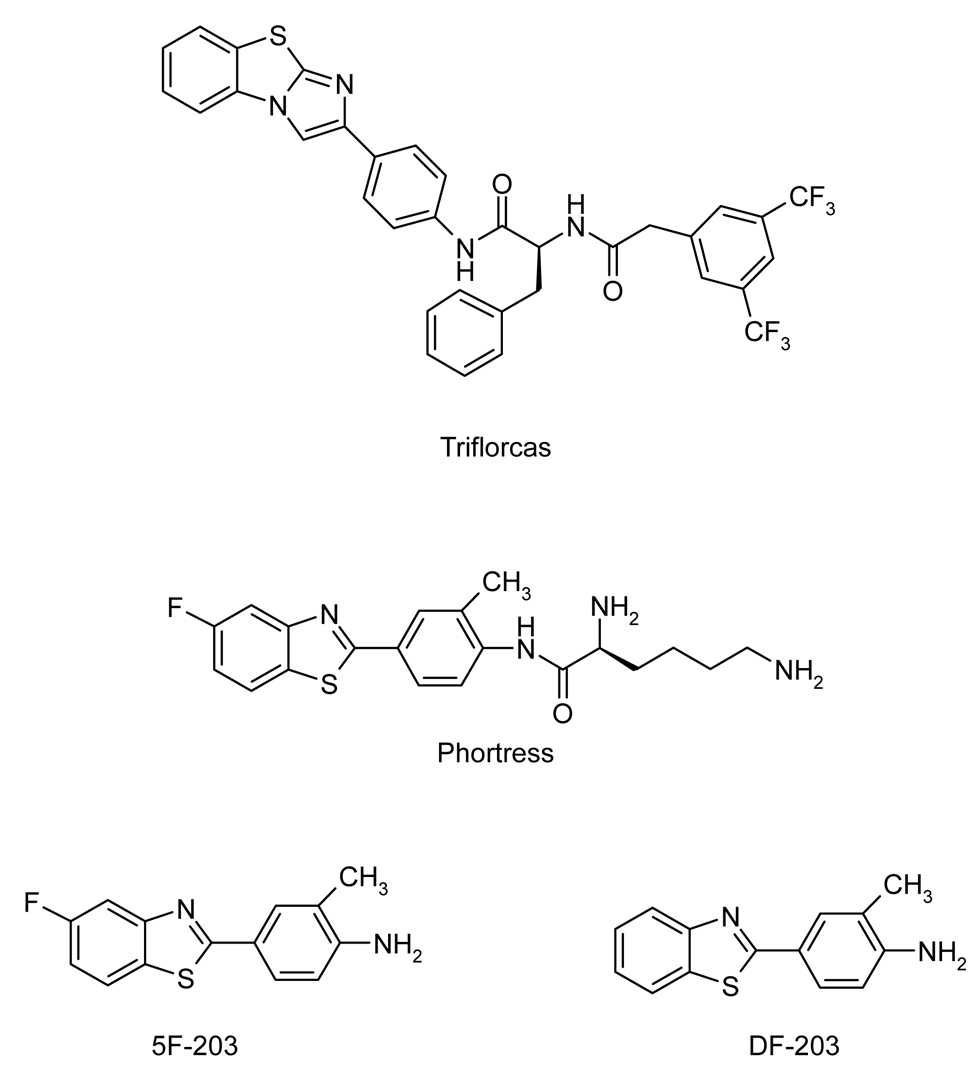

Supplement: Figure S6 — Chemical formulas of Triflorcas, Phortress, and other compounds characterized by the benzothiazole group. (TIF) [file pone.0046738.s006.tif]

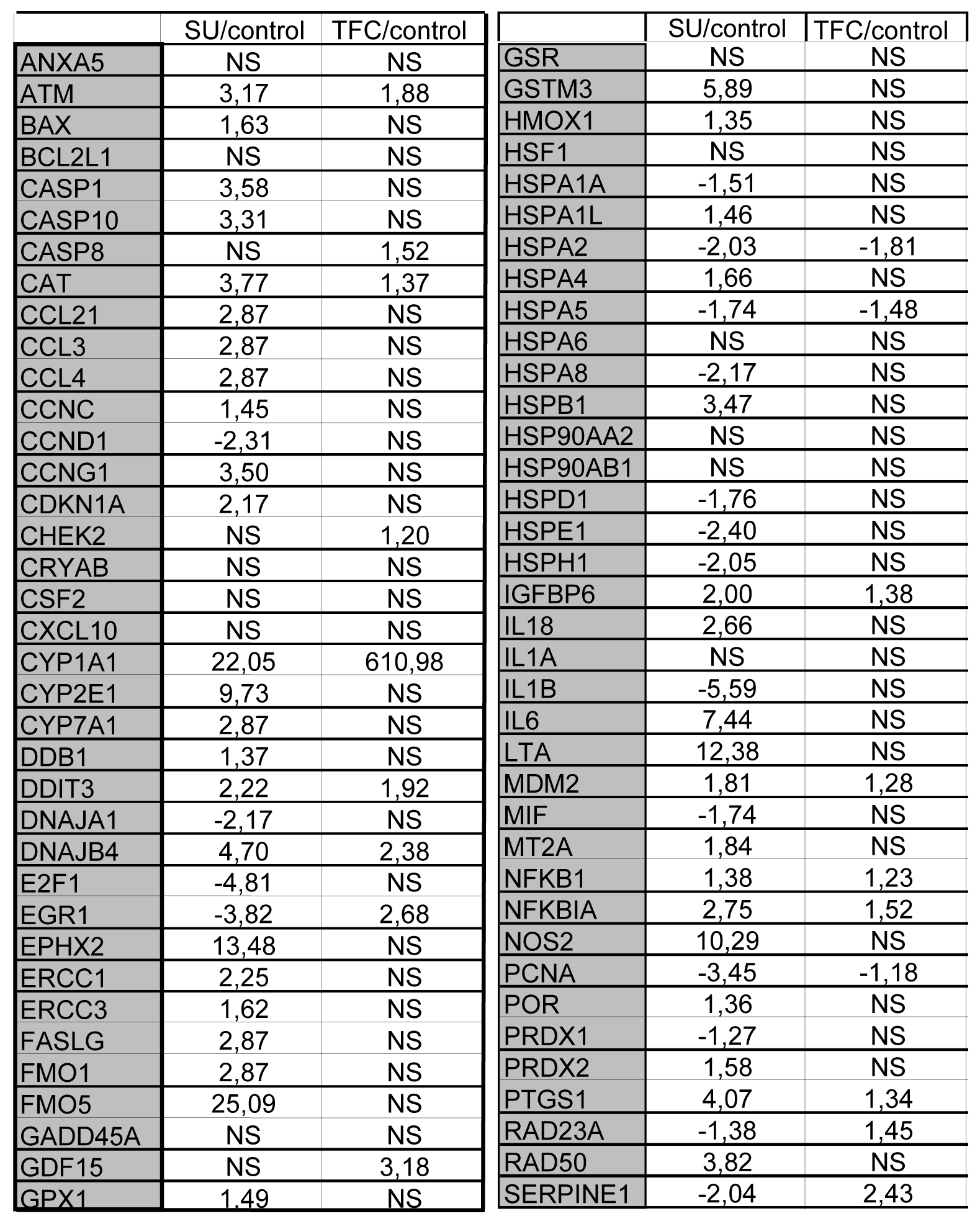

Supplement: Table S1 — Ratios of gene expression analyzed with RT-PCR stress and toxicity array. Cells were treated either with SU11274 (SU) or with Triflorcas (TFC). NS indicate not statistically significant changes. (TIF) [file pone.0046738.s007.tif]

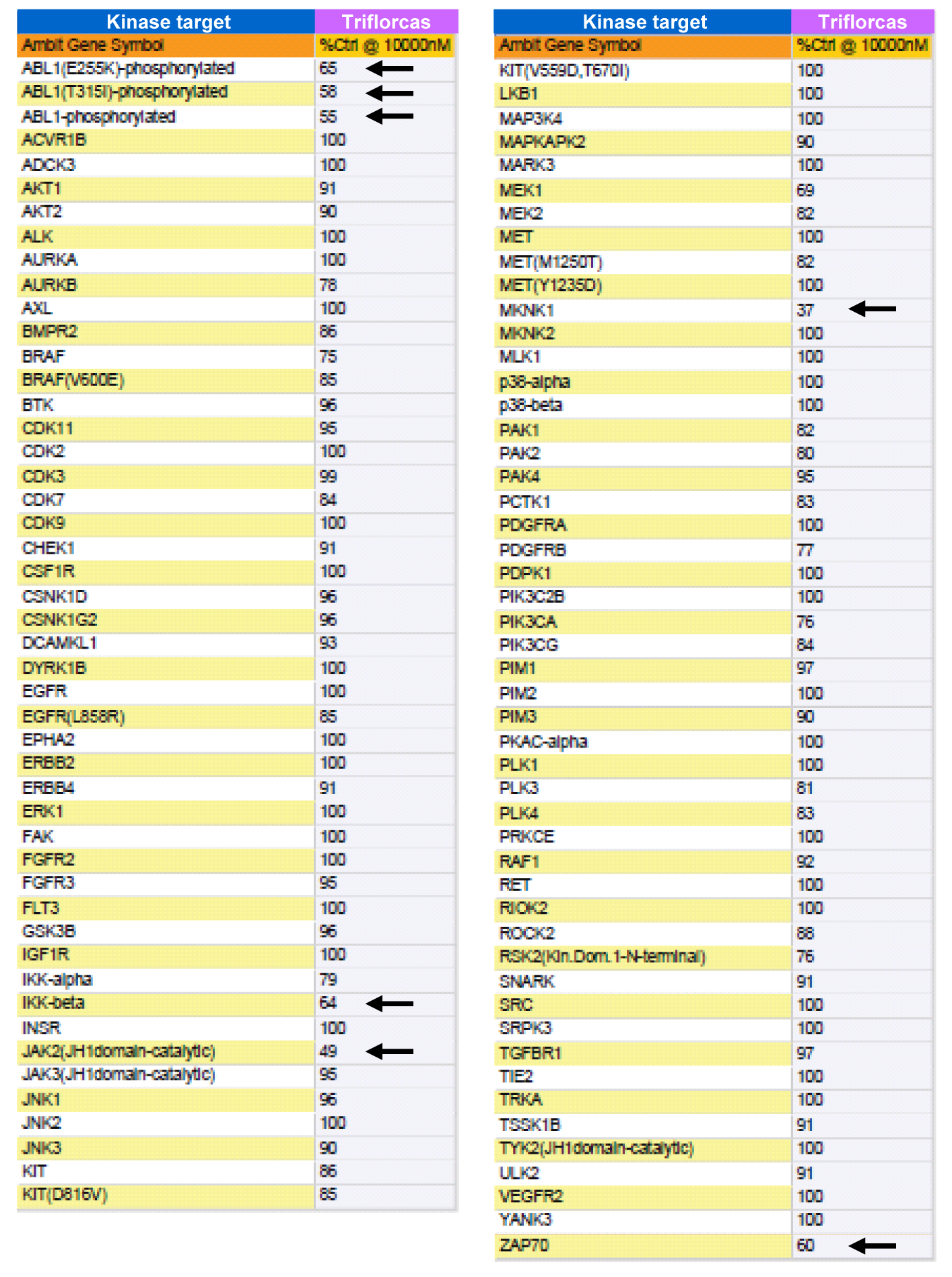

Supplement: Table S2 — Selectivity profile of Triflorcas. Triflorcas was screened against a KINOMEscan (http://www.kinomescan.com) panel of 98 kinases. The ratio of binding (% TCF over control condition) for each kinase is indicated. The arrows indicate the kinases for which Triflorcas reduced more than 30% the binding constant. (TIF) [file pone.0046738.s008.tif]

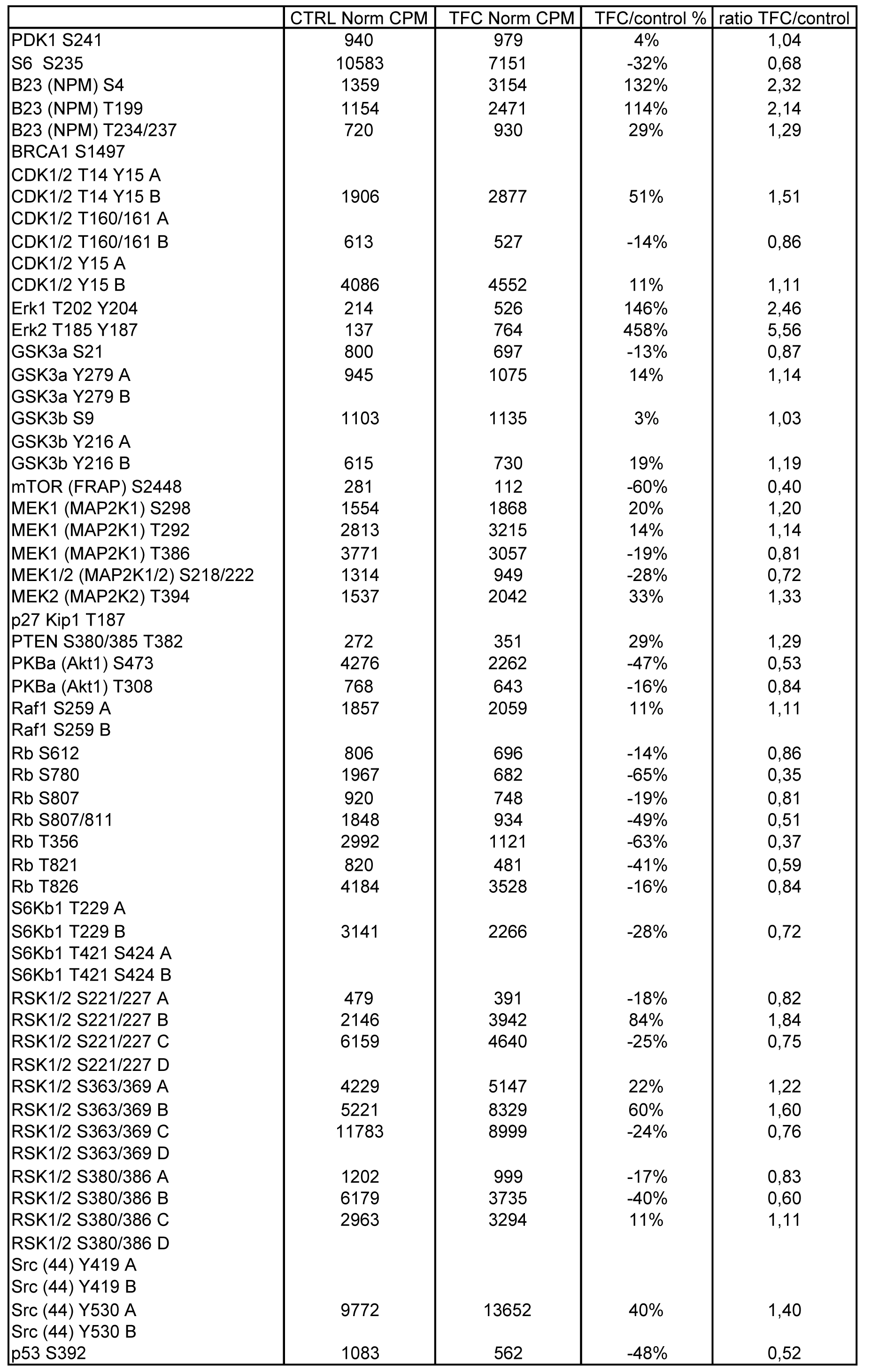

Supplement: Table S3 — Triflorcas effects on the phosphorylation status of several cell cycle proteins analyzed by the phospho-array KPSS 10.1 (Kinexus Bioinformatics). GTL-16 cells were treated with vehicle (CTRL) or Triflorcas (TFC; 3 µM) for 72 hours (first and second column, respectively). Intensities are represented as normalized counts per minute (CPM). Data for untreated or treated cells are reported. Changes in phosphorylation levels are expressed as either percentage or ratio of TFC-treated over control (third and fourth column, respectively). (TIF) [file pone.0046738.s009.tif]

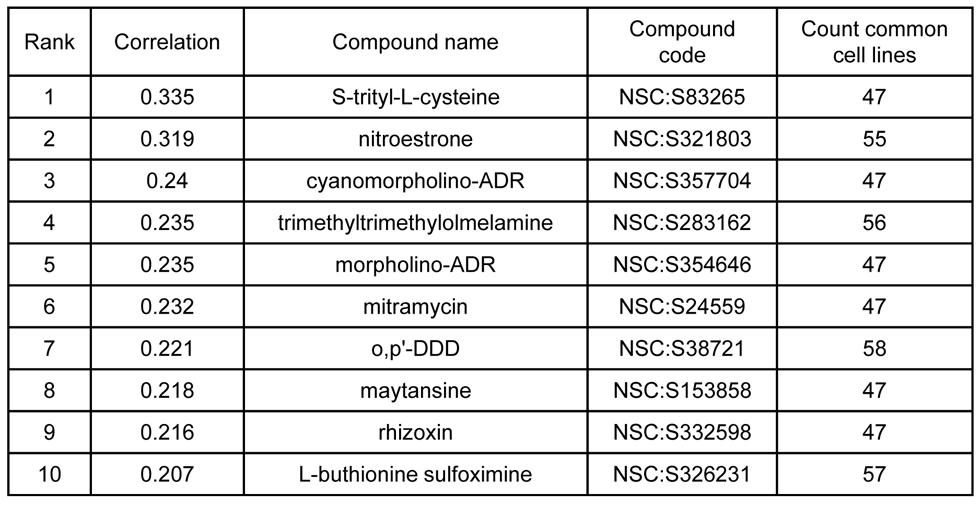

Supplement: Table S4 — Comparison of Triflorcas activity to standard agents in the NCI60 screen using the COMPARE software. The 10 drugs most similar in Triflorcas activity are reported. (TIF) [file pone.0046738.s010.tif]

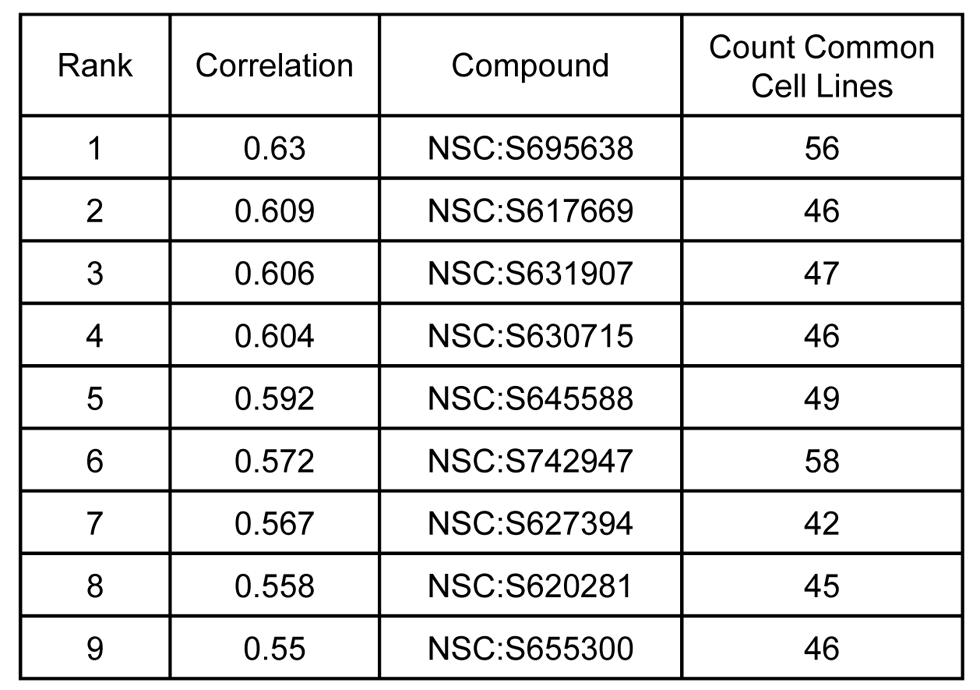

Supplement: Table S5 — Comparison of Triflorcas activity to synthetic publicly available anticancer compounds in the NCI60 screen using the COMPARE software. The 9 drugs most similar in activity are reported. (TIF) [file pone.0046738.s011.tif]
